# TiNiSi‐Type *A*LiAu (*A* = Ca, Sr, Ba, Eu, Yb) Compounds: Alternative Use of “Excess” Electrons

**DOI:** 10.1002/chem.202500134

**Published:** 2025-04-07

**Authors:** Peter Höhn, Daniel Menéndez Crespo, Matej Bobnar, Primož Koželj, Mitja Krnel, Yurii Prots, Marcus Schmidt, Frank R. Wagner, Yuri Grin

**Affiliations:** ^1^ Max‐Planck‐Institut für Chemische Physik fester Stoffe Chemische Metallkunde Nöthnitzer Str. 40 Dresden Germany; ^2^ Department of Condensed Matter Physics Jožef Stefan Institute Jamova cesta 39 Ljubljana Slovenia; ^3^ Department of Physics, Faculty of Mathematics and Physics University of Ljubljana Ljubljana Slovenia

**Keywords:** auride, chemical bonding, crystal structure, electrical conductivity, magnetism, structure type TiNiSi

## Abstract

New TiNiSi‐type compounds *A*LiAu (*A =* Ca, Sr, Ba, Eu, Yb) were obtained in the form of mm‐sized single crystals by high‐temperature centrifugation‐aided filtration from lithium melt. They are the first examples of TiNiSi‐type representatives containing Li and a transition metal. The metallic phases show paramagnetic (Ca) or diamagnetic (Sr, Ba, Yb) behavior or antiferromagnetic ordering below 19 K (Eu). A new structural description is based on a hexagonal close packing with *A* occupying all octahedral and Li occupying half of the tetrahedral voids in an ordered fashion. Chemical bonding analysis supports the structural description and reveals the formation of eight‐atomic *stella–quadrangula* bonds pinned on the empty tetrahedral holes, a bonding picture known from elemental metals.

## Graphical abstract

Excess electrons distinguish the bonding in *A*LiAu (*A* = Yb, Ca, Eu, Sr, Ba), a new series of intermetallic compounds crystallizing in TiNiSi type. According to bonding analysis, these metallic phases are classified as aurides containing polycations with multiatomic bonding between *A*, Li, and Au

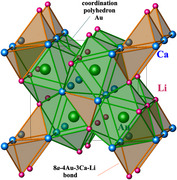

## 1 Introduction

Compounds with a low number of valence electrons often show multi‐center bonding and exhibit metallic behavior. The TiNiSi type of structure (space group *Pnma*, *oP*12, #62), a crystallographically ordered variant of the PbCl_2_ family, has several hundred representatives ranging from ionic salts to intermetallic phases,^[^ ^1^ ^]^ providing a playground for the investigation of structure‐bonding relationships due to the wide range of valence electron counts (VEC).^[^ ^2^, ^3^, ^4^ ^]^ Alkali and alkaline‐earth elements cause clearly defined VEC in these phases. The non‐metallic pnictogenide Zintl phases *A*Li*E* (*A* = Ca, Sr, Eu, Yb; *E* = As, Sb, Bi) are up to now the only phases reported to contain both an alkali and an alkaline‐earth element.^[^ ^5^, ^6^ ^]^ The unique properties of gold to adopt a negative charge led us to investigate the corresponding systems containing late transition metals instead of pnictides.

A wide range of binary phases *A*─Au with stoichiometry from 7:3 to 1:4 and Li─Au (15:4–1:3) is well investigated with Au always acting as anion.^[^ ^7^, ^8^, ^9^, ^10^, ^11^ ^]^ However, no ternary phases *A*─Li─Au have been reported up to now. Compared to phases *A*LiAu, the compounds Yb_2_Au,^[^ ^12^ ^]^ BaMgAu,^[^ ^13^ ^]^ YbMgAu,^[^ ^14^ ^]^ and EuMgAu^[^ ^14^ ^]^ also crystallize in the TiNiSi type structure, but contain one valence electron more per formula unit (Li: 1*e*, *A*: 2*e*, Eu[4f^7^]: 2*e*, Yb[4f^14^]: 2*e*, Au[5d^10^]: 1*e*).

## 2 Results and Discussion

### 2.1 Synthesis

**Figure 1:**
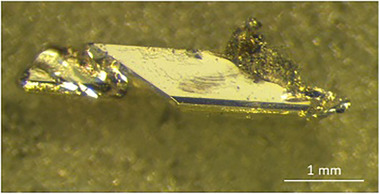
Single crystal of CaLiAu with polycrystalline adherents prepared by high‐temperature centrifugation‐aided filtration (HTCAF).

High‐temperature centrifugation‐aided filtration (HTCAF)^[^ ^15^, ^16^, ^17^ ^]^ is shown to be a powerful tool to grow larger single crystals (Figure 1) of all phases *A*LiAu (*A* = Ca, Sr, Ba, Eu, Yb). Lithium forms a highly reactive melt above 150 °C, so that synthesis and crystal growth succeed in a temperature range between 800 and 300 °C with small amounts of Li_3_Au and Li_15_Au_4_ as side products.^[^ ^9^ ^]^ The lithium melt also acts as a sink for impurities (H, N), which are bound as Li_3_N, Li_4_NH, or – due to reaction with the crucible material – Li_7_[TaN_4_].^[^ ^18^ ^]^

### 2.2 Differential Thermal Analysis

**Figure 2:**
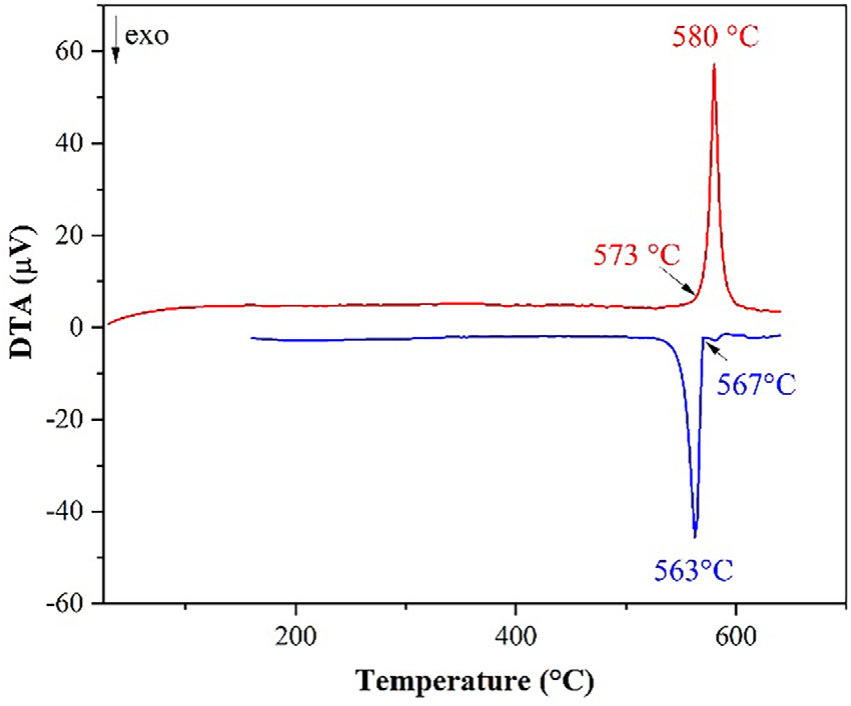
Thermal behavior of CaLiAu.

All measured TiNiSi‐type materials *A*LiAu (*A* = Ca (Figure 2), Sr, Ba, Eu, Yb) show no significant change in mass as a function of temperature in the temperature range below 700 °C. It can be assumed that none of the components is released via the gas phase, and thus the respective overall chemical composition remains constant during the measurement. For all compounds, the DTA signal of the heating curves shows in each case only a clearly defined endothermic peak (decomposition onset temperature) at 573 °C (Ca), 533 °C (Sr), 378 °C (Ba), 560 °C (Eu), and 575 °C (Yb). The decomposition temperature decreases with increasing size of the alkaline earth element (Yb^2+^ 102 pm, Ca^2+^ 100 pm, Eu^2+^ 117 pm, Sr^2+^ 118 pm, Ba^2+^ 135 pm).^[^ ^19^ ^]^

The cooling curves of all compounds show, in addition to one strong exothermic signal, smaller exothermic signals. In the case of CaLiAu, X‐ray powder diffraction data of the products show two phases, CaLiAu and CaAu.^[^ ^20^ ^]^ This behavior most probably results from peritectic decomposition, however, reaction with the crucible material cannot be ruled out as well.

### 2.3 Crystal Structure

**Figure 3:**
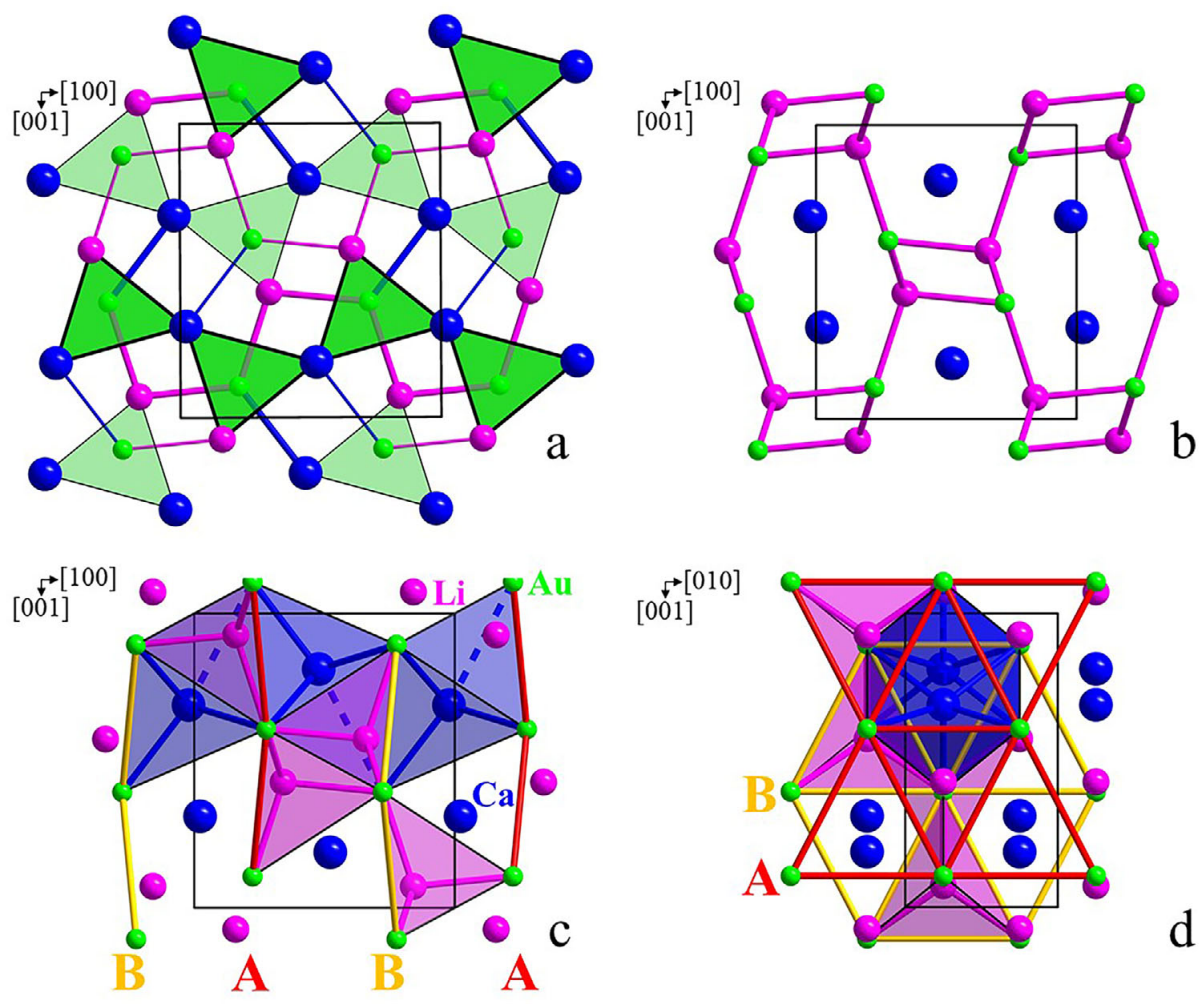
Representation of the crystal structure of CaLiAu (TiNiSi type) as a stacking of 2D walls of AuLi_2/2_Ca_4/4_ trigonal prisms (a), as a 3D polyanion [LiAu] with embedded Ca cations (b), and as closest packing *hcp* of Au (red **A** and yellow **B** nets in (100)) with octahedral (5+1) and tetrahedral coordination of Ca and Li, respectively, in its voids (c,d).

All phases *A*LiAu (*A =* Ca, Sr, Ba, Eu, Yb) crystallize in the TiNiSi‐type structure.^[^ ^21^ ^]^ Crystallographic data, atomic coordinates, as well as isotropic and anisotropic displacement parameters (single crystal X‐ray diffraction data) are summarized in Tables S1, S2, and S3, Supporting Information, respectively; relevant distances are listed in Table S4, Supporting Information. The crystal structures of the TiNiSi type (schematically *AA*′*E*) are described in the literature either crystallographically as chains of side‐edge‐condensed columns of trigonal prisms [*EA*_4_ *A*′_2_] forming on this way walls parallel to [100]; neighboring walls are shifted along [010] by *b*/2 (Figure 3a),^[^ ^14^ ^]^ or as a tridimensional polyanion [*A*′*E*] with the cations *A* embedded in its cavities, assuming charge transfer from cations to the polyanion (Figure 3b).^[^ ^22^ ^]^ Recent detailed analysis of chemical bonding does not support the second representation.^[^ ^4^ ^]^ More rare is a representation in form of nearly closest 3^6^ layers of gold stacked along [010] according to the hexagonal closest packing.^[^ ^23^ ^]^ Half the tetrahedral voids are occupied by Li and the octahedral voids are occupied by Ca (Figure 3c,d and Figure S1, Supporting Information).

The lattice parameters of *A*LiAu compounds investigated increase rather isotropically with the size of the *A* component represented by the effective ionic radius (Figure S2, Supporting Information) starting with the smallest representative Ca and increasing in the sequence Yb, Eu, Sr, and Ba,^[^ ^19^ ^]^ whereas the trend of the interatomic distances shows significant differences depending on element combination and crystallographic direction (Figure S3, Supporting Information). So, all “short” distances *A*─Au (300 pm < *d* < 350 pm) increase relative to the changes in atom radius (*d*_ave_) and unit cell size (*V*), whereas the longest distance *A─*Au (*d*_long_) remains rather constant. The same is true for the Li–Au partial structure: the Li–Au distances in (101) plane increase with increasing *A* size (considering the large deviation of Li in BaLiAu compared to the other phases, the correctness of the refined Li position in BaLiAu might be questionable), whereas the Li–Au distances along [010] remain constant. The bond angles change differently in different directions in order to accommodate the larger cations (Figure S3, Supporting Information).

### 2.4 Structural Considerations

**Figure 4:**
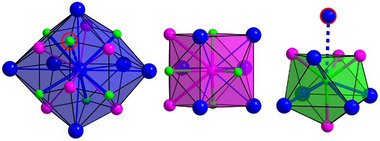
Coordination of atomic species in CaLiAu: (left) Ca, (middle) Li, (right) Au (Ca: blue, Li: pink, Au: green). The red circles around Au in the left panel and around Ca in the right panel refer to the longest Ca─Au distance to the central atom, which is, after BS, included into the coordination sphere of the Ca, but not for Au.

The characteristic behavior of the distances and bond angles led us to investigate the coordination spheres of the constituting species of CaLiAu in detail using the Brunner–Schwarzenbach (BS) approach,^[^ ^24^ ^]^ a maximum‐gap procedure to evaluate the coordination number on base of the interatomic distances. Characteristically for the TiNiSi family,^[^ ^4^ ^]^ the coordination polyhedra around Ca, Li, and Au in CaLiAu are formed by 18 (6×Ca, 6×Li, 6×Au), 12 (6×Ca, 2×Li, 4×Au) and 9 (5×Ca, 4×Li) atoms, respectively (Figure 4). It is interesting to note, that the longest Ca─Au distance considered in the polyhedron around Ca (red circle in left panel in Figure 4) does not belong to the first coordination sphere of Au, lying far *outside* the relevant range (red circle in right panel in Figure 4). The same applies for the other compounds *A*LiAu (*A =* Yb, Eu, Sr, Ba, Figure S4, Supporting Information).

Therefore, the coordination polyhedron around Au has to be regarded as a (distorted) capped square antiprism. Furthermore, it should be emphasized, that in isotypic semiconducting CaLiSb, as well as in all other *A*Li*E* (*A* = Ca, Sr, Eu, Yb; *E* = As, Sb, Bi), the coordination polyhedra and Brunner–Schwarzenbach plots show subtle, but significant differences compared to CaLiAu (see Figure S4, Supporting Information).

### 2.5 Electronic Structure and Chemical Bonding

Early discovery of CsAu^[^ ^9^ ^]^ and RbAu^[^ ^10^, ^11^ ^]^ revealed compounds, whose composition and crystal structure of the CsCl type can be easily understood assuming the charge transfer from the alkali metal to gold according to the balance Rb^1+^Au^1‒^. The latter was confirmed by DFT calculations and analysis of electron density (Rb^0.7+^Au^0.7‒^).^[^ ^25^ ^]^ From this point of view, the existence of CaLiAu looks rather unusual due to the larger number of electrons available for charge transfer and rather insufficient reduction power of Ca in comparison with Rb and Cs to achieve lower oxidation state of Au in comparison with CsAu and RbAu. Therefore, the electronic structure of CaLiAu was in focus of the study first.

**Figure 5:**
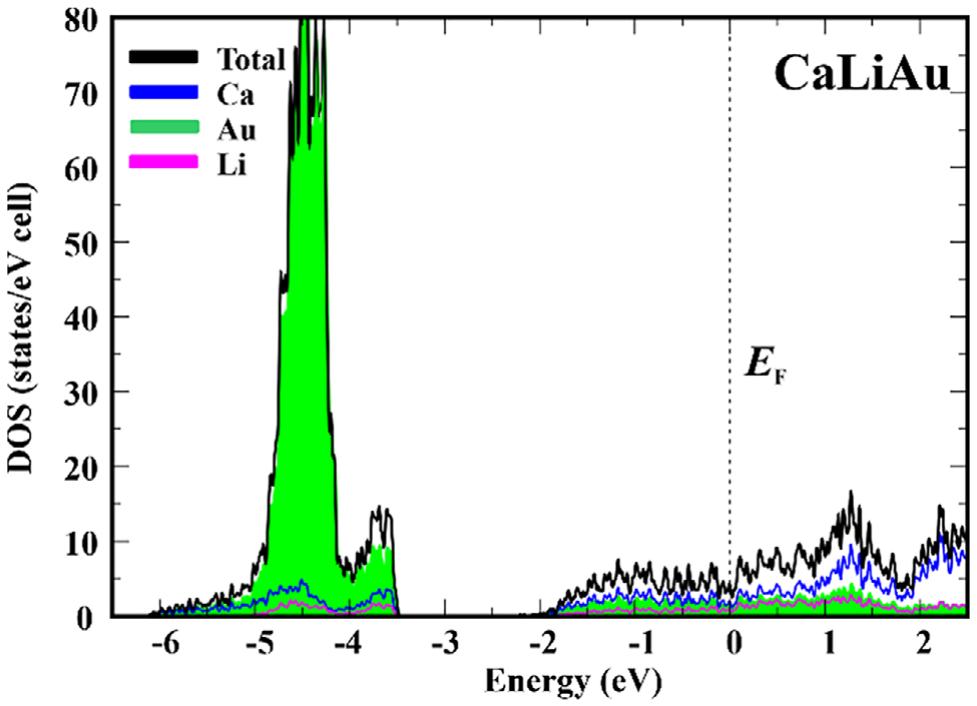
Calculated total electronic density of states (DOS) for CaLiAu together with the contributions of the three components.

Despite the fact, that CaLiAu contains three metals, the calculated electronic density of states for this compound (Figure 5, for the underlying band structure cf. Figure S6, Supporting Information) is quite clearly structured. It can be divided into two regions. The first one (−6.2 eV < *E* < −3.5 eV) is mainly built by the atomic states of gold with minor contributions of Ca and Li. The part corresponds to a nominal Au(5d^10^6s2) configuration with a conceptual charge assignment according to Au^1−^. The striking feature is the separation of this region toward higher energy by a gap of approx. 1.6 eV. The parabolic shape of the region below *E*_F_ and the non‐appearance of a gap at *E*_F_ resembles the free‐electron‐like DOS in elemental alkali metals and Al. The Total DOS in CaLiAu resembles the situation in CsAu, where the subsequent DOS region above the gap is unoccupied. In this sense, CaLiAu can be tentatively formulated as an excess‐electron compound according to Ca^2+^(Li^1+^Au^1−^)×2e^−^.

It is now interesting to analyze in more detail what happens with the “excess electrons” in this compound. In general, “excess” electrons in respect to distinct conceptual electron counting scheme, e.g. Zintl‐like,^[^ ^26^ ^]^ i.e., occupying the states above the gap, can be used either for formation of the lone pairs on cations or for making bonds between the cations, i.e., leading to the appearance of a polycation.^[^ ^26^ ^]^ Integration of the states above the gap and below the Fermi level (−1.9 eV < *E* < *E*_F_) yields 8 “excess” electrons per unit cell, i.e., two excess electrons per f.u. From the two electrons, gold contributes 1.47, Ca −0.43, and lithium −0.10 e^–^. This is comparable with the situation in lutetium monogermanide: Lu^3+^(2b)Ge^2‒^ ×1e^‒ [^ ^26^ ^]^ and points out toward possible classification of CaLiAu as an excess‐electron compound.

The possible reasons for such observations were obtained from the analysis of chemical bonding in position space applying the electron localizability approach.^[^ ^27^, ^28^ ^]^ First the atomic regions were defined using zero‐flux points in the distribution of the electron density. These regions represent atoms in the frame of the Quantum Theory of Atoms in Molecules.^[^ ^29^ ^]^ The full (total) electron density in CaLiAu does not reveal any non‐nuclear maxima, clearly ruling out the interpretation as “electride”.

**Figure 6:**
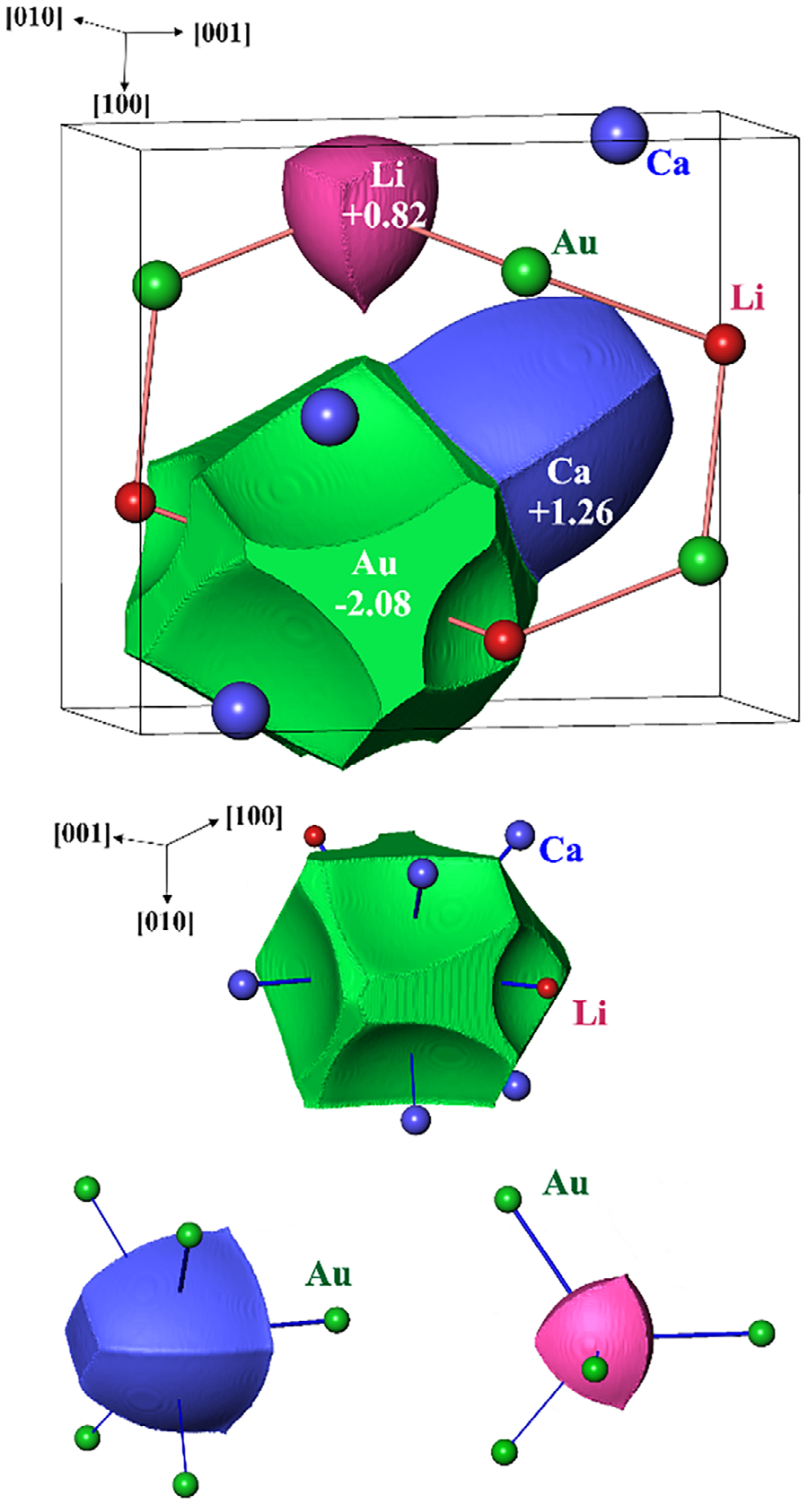
Electron density analysis in CaLiAu: (top) Atomic shapes and effective charges of the components in CaLiAu. (bottom) Topological coordination of atoms based on contact surfaces between the QTAIM atomic shapes (green – Au, blue – Ca, pink – Li).

Integration of electron density within these regions results in their effective populations. The so‐obtained values reveal clear anionic character of gold among the metallic components of this compound (Figure 6). While calcium and lithium show positive Bader charges around +1, gold reveals a large negative value, with −2.08 being essentially larger as this was expected from the electron balance above.

On the basis of the calculated electron density distribution in CaLiAu an in‐depth analysis of the coordination situation of all species can be obtained. Topological analysis of the electron density yields the QTAIM atomic domains. The solid angles subtended by the interatomic surfaces are employed to set up topological coordination numbers (*tCN*s) for each species and combine mutually consistent (with respect to coordination reciprocity) coordination situations of each species to construct coordination scenarios with variable overall weights.

The QTAIM atomic domain of Au displays 22 interatomic faces, which yields its total topological coordination number *tCN*_tot_(Au) = 22. This is much larger than nine, as indicated from the analysis of interatomic distances (Figure 4). The contributions of the ligands are classified according to the solid angle subtending the contact surface^[^ ^30^ ^]^ and result in the topological number of maximal weight *tCN*_max_(Au) = 9 (Figure 6, bottom left). The according values for Ca and Li are *tCN*_tot_(Ca) = 6, *tCN*_max_(Ca) = 5, *tCN*_tot_(Li) = *tCN*_max_(Li) = 4 (Figure 6, bottom middle and right). The coordination scenario with the highest weight^[^ ^30^ ^]^ obtained is the [Ca^[0Li,5Au;0Ca]^ Li^[0Ca,4Au;0Li]^ Au^[4Li,5Ca;0Au]^] one. This corresponds to a hetero‐ionic 9‐coordination of Au in agreement with the BS scheme. In contrast to the BS scheme, the inclusion of coordination reciprocity yields the associated 4‐ and 5‐coordinations for Li and Ca species, respectively. From the topological coordination point of view, the atomic shapes in CaLiAu (Figure 6) are similar to those in SrLiAs^[^ ^30^ ^]^ rather than to those in the structure‐type eponym TiNiSi and other representatives like Co_2_Si.^[^ ^30^ ^]^

Further information about the chemical bonding between its actors – atoms – is obtained from the combined analysis of electron density and electron‐localizability indicator (ELI in the ELI‐D representation). While the distribution of ELI‐D around free atoms is spherical, in crystal structures it becomes structured – as a rule in the valence region, and – in some cases – in the region of the penultimate shells as well.^[^ ^27^, ^28^ ^]^ The local maxima of ELI‐D in the valence region visualize different types of bonds. Integration of electron density within the bond basins yields their electronic populations. Intersection analysis of the ELI‐D/QTAIM basins results in the total and the effective bond basin atomicity, i.e., the number of atoms that significantly contribute to the bond basin population. Integration of electron density in the bond basin yields their populations.^[^ ^27^, ^28^ ^]^

**Figure 7:**
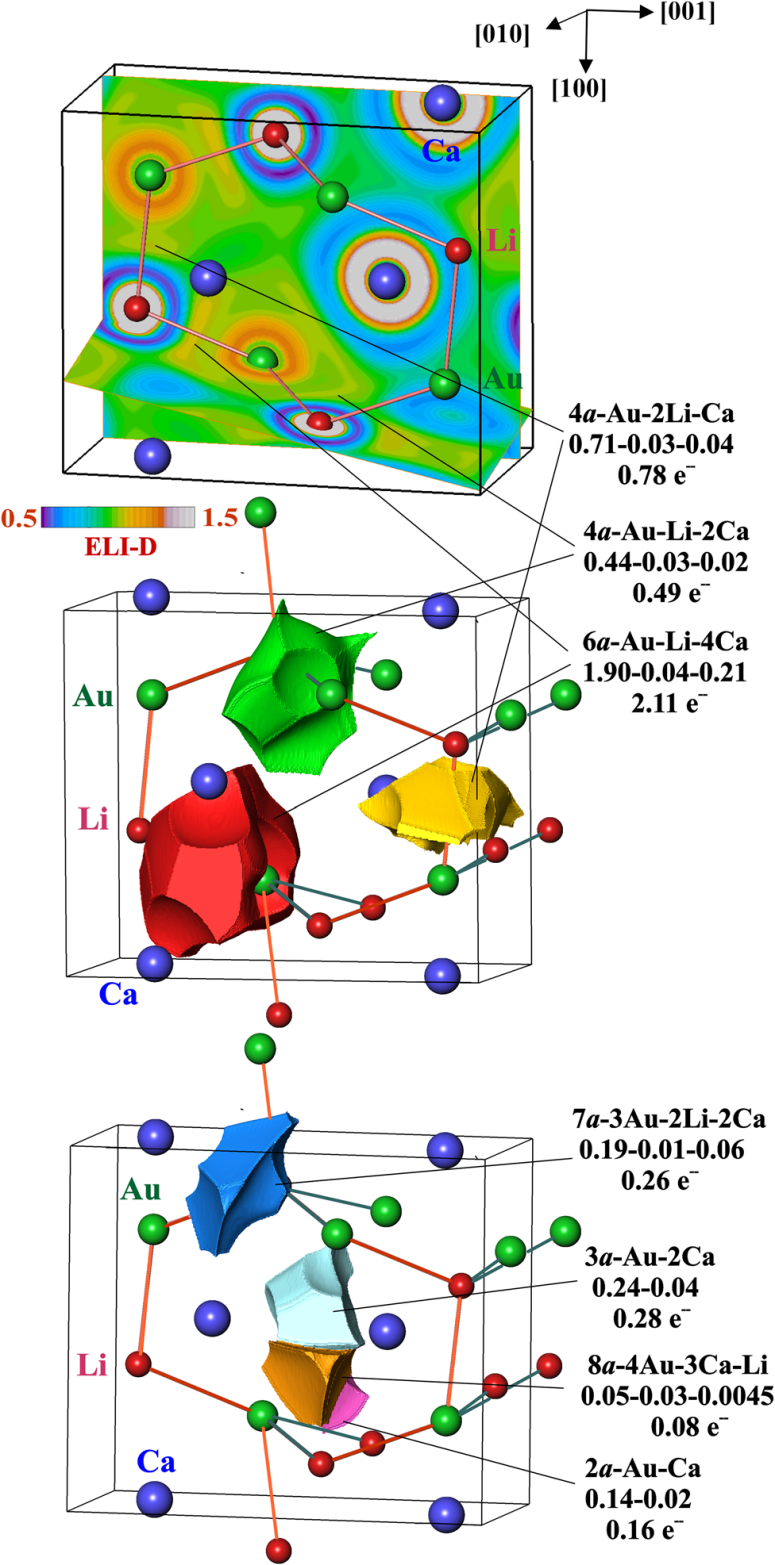
Electron localizability indicator in CaLiAu: (top) ELI‐D distribution in the planes of Li─Au contacts; (middle) bond basins on the Li─Au contacts (higher populations); (bottom) bond basins located out of the Li─Au contacts (lower populations).

In the case of CaLiAu, this analysis shows, that the bonding between the atoms typically does involve more than two atoms (unlike in the classical 2‐center bonding scenario) for virtually all kinds of short interatomic contacts, beside one two‐atomic Ca─Au bond with quite low population of 0.16 e^‒^ (Figure 7, middle and bottom). The ELI‐D maxima located on the shortest Li─Au contacts (Figure 7, top), which in the two‐dimensional maps look like two‐atomic Li‐Au bonds, may represent the formation of a Li─Au polyanion. De facto, their basins are 4‐ and 6‐atomic with essential contribution of the calcium atoms bridging the Li─Au contacts (Figure 7, top and middle). Analysis of their basin populations reveals, that their major contributions (89–91%) originate from the gold species (Figure 7, middle). Thus, they represent multiatomic strongly polar‐to‐ionic bonding, agreeing with the higher covalency of the Ca─Au in comparison with Li─Au found for CaLi*E* compounds.^[^ ^4^ ^]^ This rules out the bonding interpretation in terms of a Li─Au polyanion. All other bond basins with the participation of gold follow this trend. Here, gold contributes 73–88% of the bond populations (Figure 7, bottom).

**Figure 8:**
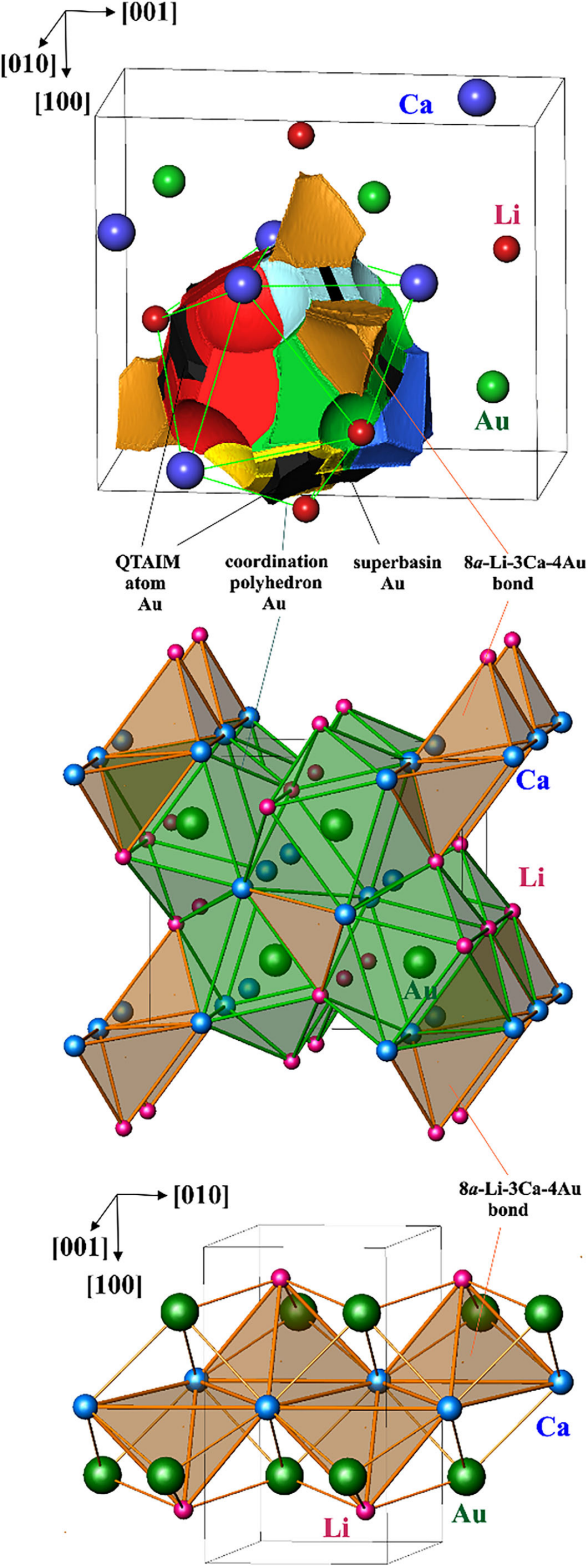
Bonding picture in CaLiAu: (top) bond basins with participation of gold (superbasin Au multi‐color) in comparison with the QTAIM gold atom (black); (middle) Au‐based substructure shown by the coordination polyhedrons of gold (green) interconnected by 8*a*‐4Au─3Ca─Li bonds (central part (Li─3Ca) is shown in orange in middle and bottom panels) centered in the tetrahedral holes between the [AuCa_6_Li_4_] polyhedrons; (bottom) chain of *stella–quadrangula* 8*a*‐4Au─3Ca─Li bond units along the [010] direction. Color code for bond basins is the same as in Figure 7.

There is only one bond basin in CaLiAu, which is located between the QTAIM gold atoms: 8*a*‐4Au─3Ca─Li, the *stella–quandrangula* bond (light brown in Figures 7, bottom and 8, top). It represents an eight‐atomic bond and visualizes the use of the “excess” electrons for formation of the multiatomic bonds involving both, cationic and anionic, components. Such bonds, centered at the tetrahedral holes, are part of the bonding picture in elemental metals^[^ ^31^, ^32^, ^33^, ^34^ ^]^ and compounds based on the closest packings, like Be_3_Ru.^[^ ^35^ ^]^ The new feature of the *stella–quadrangula* bond, reflected in its designation, is the participation not only of the atoms forming the inner tetrahedron (hole), like in Be_3_Ru and in elemental metals, but also the atoms of the outer tetrahedron. Such tetrahedral *stella–quadrangula* bonds form chains along [010] (orange tetrahedra in Figure 8, middle and bottom showing the central LiCa_3_ parts). Even if on the calculational level, the population of this basin is low (0.08 e^‒^), the appearance of this bond agrees with the occupation of the states after the gap in the electronic DOS (cf. above).

All basins, where gold is involved, are nearly completely embedded in the QTAIM atom of gold (black in Figure 8, top) or in the coordination polyhedron of Au (thin green lines in Figure 8, top). The packing of the [AuLi_4_Ca_6_] polyhedra represents the main part of the CaLiAu structure (green polyhedra in Figure 8, middle). The *stella–quadrangula* multicenter bonds are embedded in‐between. This picture confirms the classification of CaLiAu as a system with “excess” electrons. It completes the sequence with the other known representatives of this family. While in Ga_2_Se_2_ the polycation formed by means of “excess” electrons appears in a shape of a two‐atomic dumbbell [Ga_2_], in LuGe it is an isolated tetrahedron [Lu_4_], in CaLiAu a new way of employing “excess” electrons is found. They are used for multi‐atomic bonds involving the cationic and anionic components.

### 2.6 Hydrogen Stabilization?

The interesting feature of ELI (and – earlier – of the electron localization function ELF), besides being a powerful quantum chemical technique for chemical bonding analysis, the electron‐localizability indicator visualizes regions in crystal structures suitable for localization of anions, in particular the hydride one.^[^ ^36^, ^37^, ^38^, ^39^, ^40^ ^]^ That means that the ELI‐D maximum representing the eight‐atomic 4Au–3Ca–Li bond may also hint to the possible position of the hydridic hydrogen.

To evaluate the possibility of hydrogen stabilization of CaLiAu, the schematic equilibrium

$$\begin{array}{c} \text{CaLiAu}+\frac{1}{2}H_{2}↔\text{CaLiAuH} \end{array}$$

was investigated from the point of view of total energy. For this purpose, first the quantum mechanical optimization of the crystal structures (using the experimental symmetry and lattice parameters) was applied for the localization of the hydrogen in the atomic arrangement (the refinement using even the single crystal X‐ray diffraction data was not possible). The optimized atomic coordinates were used for further calculations. The total energy of the left part is smaller by 0.0007 Ha = 1.8 kJ mol^−1^. The small value indicates that CaLiAu should be more stable than the hydride variety, however, the formation of the hydride cannot be completely excluded by theoretical techniques employed. Therefore, a further series of three experiments (H, N, N+H) was focused on the possibility of introduction of hydride and/or nitride ions in the single tetrahedral void of the TiNiSi type structure (0.125 g Li, 0.102 g Ca, 0.520 g Au, 0.007 g LiH, 0.013 g Li_3_N) no indications for the inclusion of H or N and the formation of a quaternary phase were observed. TG‐MS measurements on selected single crystals showed H concentrations – if any – at the detection limit corresponding to CaLiAuH_<0.01_.

### 2.7 Magnetic Susceptibility and Electrical Conductivity

**Figure 9:**
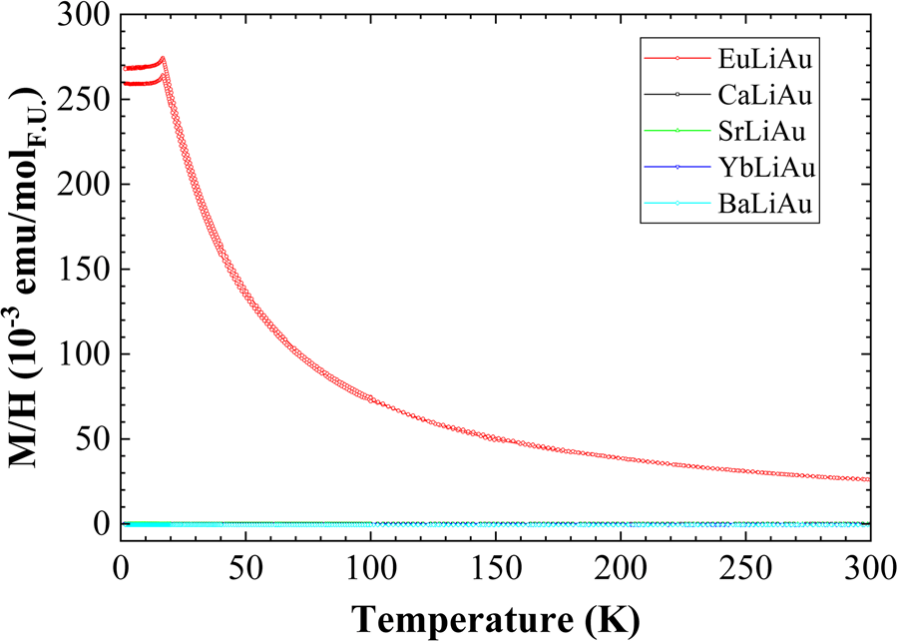
Magnetic susceptibility of *A*LiAu (*A* = Ca, Sr, Ba, Eu, Yb); magnetic field *μ*_0_ *H* = 7 T.

EuLiAu shows antiferromagnetic ordering below 19 K and Curie‐Weiss‐type paramagnetism above (Figure 9). At temperatures above 40 K, the susceptibility obtained at 0.01 T was fit to the Curie‐Weiss law, giving an effective magnetic moment of 6.63(3) *μ*_B_ / f.u. and the Curie‐Weiss temperature *Θ*_CW_ = −8.4(5) K. The magnetic moment is a bit lower than the theoretical value of 7.94 *μ*_B_ for the Eu^2+^ ion. The magnetization *M*(*T*) of the other *A*LiAu (*A =* Ca, Sr, Ba, Yb) samples, reveals that CaLiAu is paramagnetic, whereas the other three samples are diamagnetic (Figure 9).

In agreement with the calculated electronic DOS, the electrical resistivity *ρ*(*T*) measured on crystals of CaLiAu, SrLiAu, BaLiAu, YbLiAu, and EuLiAu (Figure S7) shows for all compounds metallic behavior with the resistivity values at 300 K (*ρ*_300K_) of 19±1, 29±2, 21 570±100, 17 160±80, and 56±3 µΩ cm and residual resistivity ratios (RRR = *ρ*_300K_/*ρ*_4K_) of 85, 15, 1.3, 2.4, and 7.5, respectively. The resistivity measurements for CaLiAu, SrLiAu, BaLiAu, and YbLiAu do not show any phase transitions or superconductivity above 4 K. The resistivity of EuLiAu has a *T* ^5/2^ type temperature dependence for temperatures below 19 K, in accordance with the antiferromagnetic ordering, and linear temperature dependence above.

## 3 Conclusions

The new family of TiNiSi‐type phases *A*LiAu (*A* = Yb, Ca, Eu, Sr, Ba), have been prepared by HTCAF in form of mm‐sized single crystals. Their crystal structures are best described as a *hcp* arrangement of Au with octahedral voids occupied by *A* and half of the tetrahedral voids filled by Li. According to bonding analysis by quantum chemical techniques in position space, this structural description is confirmed and eight‐atomic *stella–quadrangula* bonds centered at the empty tetrahedral holes of the hcp Au arrangement were found. This bonding picture resembles one in elemental metals and intermetallic compounds showing closest packings of atoms. It illustrates an alternative way of use of “excess” electrons for stabilization of structural patterns.

## 4 Experimental Section

### Synthesis

In view of the sensitivity of the starting materials and the reaction products to moisture and air, all manipulations associated with sample preparation and handling were performed in inert atmosphere using an argon‐filled (Praxair, >99.999%, purified with BTS catalyst) glove box (MBraun, *p*(O_2_)/*p*_0_ < 0.1 ppm, *p*(H_2_O)/*p*_0_ < 0.1 ppm). The elements Li (Evochem, 99.9%), Ca (Alfa, dendritic pieces, 99.987%), Sr (Alfa, dendritic pieces, 99.9%), Ba (Alfa, dendritic pieces, 99.9%), Eu (Hunan Rare Earth Metals Materials, pieces, 99.9%), Yb (Hunan Rare Earth Metals Materials, pieces, 99.9%), and Au (Alfa, powder, 99.998%) were used without further purification. Li_3_N was synthesized from elemental Li and nitrogen (Praxair, 99.999%, further purified by molecular sieve, 3 Å, Merck, and BTS catalyst) at 573 K.

Sample preparation was carried out employing a modified high temperature centrifugation‐aided filtration (HTCAF) technique.^[^ ^17^ ^]^ The materials were placed in a Ta ampoule (Plansee) equipped with a strainer, welded shut, and subsequently sealed in a quartz tube. The quartz tube was placed in a stainless‐steel container fitted with quartz wool and put in a box furnace. The temperature program involved heating to 1073 K by 100 Kh^−1^, annealing for 2 h and subsequent cooling with 0.5 Kh^−1^ to 573 K for the Ca, Sr and Ba, and with 1 Kh^−1^ to 523 K for the Eu and Yb phases, respectively. After turning the stainless‐steel container by 180°, the sample was centrifuged at 3000 min^−1^, removing the flux consisting mostly of lithium and further unspecified impurities.

In all experiments, agglomerates of silverish‐black pseudo‐tetragonal prisms of *A*LiAu up to several mm in size, as well as differing, but small amounts of Li_3_Au and Li_15_Au_4_ were obtained from mixtures of 0.098 g Li, 0.084 g Ca/0.183 g Sr/0.289 g Ba/0.320 g Eu/0.363 g Yb and 0.5 g Au corresponding to a molar ratio Li: *Ae*: Au ∼ 28: 4: 5. The solidified flux was not further investigated.

In contrast, in corresponding experiments with magnesium, lithium and gold, mixtures of the Heusler phase Li_2_MgAu^[^ ^41^ ^]^ and the binary MgAu^[^ ^42^ ^]^ were obtained.

In a second series of experiments aimed at phases containing both Au and N and using the same starting materials upon addition of 0.013 g Li_3_N (corresponding to a molar ratio Li: *A*: Au: N ∼ 29: 4: 5: 0.3), besides small amounts of Li_3_N and EuN in the respective experiment, identical results were obtained.

### DTA/TG

The thermal behavior of *A*LiAu (*A =* Ca, Sr, Ba, Eu, Yb) was investigated using a STA 449 C (NETZSCH). The measuring systems are operated in argon atmosphere of a glove box (MBraun).

The DTA‐TG‐measurements was examined in flowing argon atmosphere (Ar 99.999% 100 mL/min with subsequent drying and oxygen post‐purification via Big Oxygen Trap by Trigon Technologies).

The individual samples were measured under following conditions – temperature range: *A*LiAu (*A =* Ca, Sr, Eu, Yb): 25 to 600 °C and BaLiAu 25 to 800 °C, sample mass: 45–97 mg, rate of heating and cooling: 10 K/min, crucible: tantalum DTA/TG crucible with perforated lid, thermocouple: type S (PtRh/Pt). The detected mass changes depending on temperature were subjected to buoyancy correction in accordance with the measurement conditions.

### X‐ray diffraction

Powder X‐ray diffraction data (Figure S8, Supporting Information) of selected and finely ground black crystals of all phases were collected on a Huber G670 imaging plate Guinier camera (2*Θ*_max_ = 100°) using a curved germanium (111) monochromator and Cu‐K_α1_ (*A =* Ca, Sr, Ba, Yb) or Co‐K_α1_ radiation (*A* = eu) at 293(1) K. The powder samples were placed between Kapton foils to avoid degradation in air. Preliminary data processing was done using the WinXPow program package.^[^ ^43^ ^]^

After identification of known impurities (Li_3_Au, Li_15_Au_4_^[^ ^9^ ^]^), the reflections of the X‐ray powder diagrams of all samples could be indexed in the orthorhombic system (Table S1, Supporting Information). Systematic extinctions were identified leading to space group *Pnma* (#62) and its orthorhombic subgroups; all further calculations were executed in the highest‐symmetry space group. Scrutinizing the ICSD database hinted at a TiNiSi type structure.^[^ ^21^ ^]^ Subsequent refinement of X‐ray powder data of selected samples with the program packages WINCSD/JANA resulted in similar results as refinements of X‐ray data collected on single crystals.

X‐ray diffraction intensity data of single crystals of phases (*A*LiAu, *A =* Yb, Ca, Sr, Eu, Ba) sealed in glass capillaries were collected at room temperature on a Rigaku AFC7 diffractometer equipped with a Saturn 724+ CCD area detector (Mo*K*_α_ radiation). Indexing of the single‐crystal diffraction images yielded orthorhombic unit cells (Laue class *mmm*) with the lattice parameters given in Table S1, Supporting Information. Literature data^[^ ^21^ ^]^ were employed for the initial structural model, the subsequent refinement was done with the SHELXL‐2018/1 software.^[^ ^44^, ^45^ ^]^ After several cycles of least squares refinement and difference Fourier mapping, all atomic positions were found and all heavy atom positions were refined in anisotropic approximation of atomic displacement. No indications for a possible phase width or partial occupation of atomic sites were observed.

Deposition Number(s) = CSD‐2414770 (for YbLiAu), CSD‐2414848 (for CaLiAu), CSD‐2414768 (for SrLiAu), CSD‐2414769 (for EuLiAu), and CSD‐2414771 (for BaLiAu) contain(s) the supplementary crystallographic data for this paper. These data are provided free of charge by the joint Cambridge Crystallographic Data Centre and Fachinformationszentrum Karlsruhe <url href = “http://www.ccdc.cam.ac.uk/structures”>Access Structures service.

### Magnetic properties measurements

Magnetic properties measurements were conducted by a Quantum Design MPMS XL‐7 SQUID magnetometer equipped with a 7 T magnet. Magnetization was measured in the temperature range from *T* = 2 to 300 K in DC magnetic fields of *µ*_0_ *H* = 7, 3.5, 1, 0.1 and 0.01 T for all samples. The samples in crystalline form were placed in sealed evacuated quartz tubes, of which the diamagnetic background signal was subtracted. All samples contained small amounts of ferromagnetic and paramagnetic impurities. The ferromagnetic contributions to the total magnetizations were subtracted by using the Honda‐Owen extrapolation to infinite magnetic field.^[^ ^46^, ^47^ ^]^ The concentrations of iron equivalent ferromagnetic impurities were 180 ppm, 36 ppm, 3 ppm and 5 ppm, respectively. From the magnetizations, the magnetic susceptibilities *χ* = d*M*/d*H* were calculated.

### Electrical resistivity measurements

The electrical resistivity of single crystals of CaLiAu, SrLiAu, EuLiAu, and polycrystalline agglomerates of BaLiAu and YbLiAu was measured with a home‐built resistivity equipment inside a glove box, which allows for samples to be handled in an inert atmosphere. Resistivity was measured by using the standard four terminal technique. 25 µm thick Pt wires used for the terminals were contacted on the sample surfaces by using Ag epoxy paint. Resistivity was measured in the temperature range from *T* = 4 to 300 K.

### Calculation procedures

The electronic structure was calculated at the experimental geometry with FHI‐aims^[^ ^27^ ^]^ using tight settings and a GGA functional (PBE). The electronic density of states (DOS) was obtained with a PBE functional too and a mesh of 14×24×12 points. Only some small new features are incorporated into the DOS profile with respect to a mesh of 10×18×10. The number of points in the energy window was 4000. The remaining parameters are the default values in FHI‐aims. The wavefunction used for the real space bonding analysis was generated with a coarse sampling (3×6×3) of the Brillouin zone.

For the analysis of chemical bonding in position space, the electron localizability approach was utilized.^[^ ^48^ ^]^ The electron density (ED) and the electron localizability indicator (ELI‐D) were calculated, and the topological features of the computed distributions of ED and ELI‐D were analyzed with the program DGrid.^[^ ^49^ ^]^ To obtain information about the interacting atoms, the electron density was first integrated within atomic basins, i.e., spatial regions confined by zero‐flux surfaces in the gradient field of ED. This technique represents the procedure proposed in the Quantum Theory of Atoms in Molecules (QTAIM^[^ ^29^ ^]^) and provides effective electron populations for the QTAIM atoms. Further information about the bonding between atoms is obtained from a combined analysis of ED and ELI‐D.^[^ ^48^ ^]^ Solid angles subtended the faces of the QTAIM atoms, used for the evaluation of topological coordination numbers, were calculated by the program QTgeom.^[^ ^50^ ^]^

## Conflicts of Interest

The authors declare no conflicts of interest.

## Supporting information



Supporting Information

## References

[1] P. Villars , K. Cenzual , Pearson's Crystal Data: Crystal Structure Database for Inorganic Compounds, ASM International®, Materials Park, Ohio, USA 2023.

[2] W. Jeitschko , Acta. Crystallogr. B 1968, 24, 930.

[3] R. D. Hoffmann , R. Pöttgen , Z. Kristallogr. 2001, 216, 127.

[4] R. Freccero , Y.u. Grin , F. R. Wagner , Dalton Trans. 2023, 52, 8222. 37199094 10.1039/d3dt00621b

[5] X.‐J. Feng , Y.u. Prots , M. Schmidt , S. Hoffmann , I. Veremchuk , W. Schnelle , U. Burkhardt , J.‐T. Zhao , Y.u. Grin , Inorg. Chem. 2013, 52, 8971. 23863037 10.1021/ic401166v

[6] M. C. Schäfer , N.‐T. Suen , S. Bobev , Dalton Trans. 2014, 43, 16889. 25297576 10.1039/c4dt02220c

[7] F. Hensel , Z. Phys. Chem. 1987, 154, 201.

[8] U. Zachwieja , J. Alloys Compd. 1993, 199, 115.

[9] G. Kienast , J. Verma , W. Klemm , Z. Anorg. Allg. Chem. 1961, 310, 143.

[10] G. A. Tinelli , D. F. Holcomb , J. Solid State Chem. 1978, 25, 157.

[11] U. Zachwieja , Z. Anorg. Allg. Chem. 1993, 619, 1095.

[12] A. Iandelli , A. Palenzona , J. Less‐Common Met. 1969, 18, 221.

[13] M. K. Reimann , J. Kösters , R. Pöttgen , Z. Anorg. Allg. Chem. 2023, 649, e202300059.

[14] R. Pöttgen , R. D. Hoffmann , J. Renger , U. C. Rodewald , M. H. Möller , Z. Anorg. Allg. Chem. 2000, 626, 2257.

[15] Z. Fisk , J. P. Remeika , in Handbook on the Physics and Chemistry of Rare Earths, Vol. 12, Elsevier, 1989, pp. 53–70.

[16] M. Boström , S. Hovmöller , J. Alloys Compd. 2001, 314, 154.

[17] P. Höhn , J. T. Ballé , M. Fix , Y.u. Prots , A. Jesche , Inorganics 2016, 4, 42.

[18] C. Wachsmann , H. Jacobs , J. Alloys Compd. 1992, 190, 113.

[19] R. Shannon , Acta Crystallogr. A 1976, 32, 751.

[20] F. Merlo , J. Less‐Common Met. 1982, 86, 241.

[21] C. B. Shoemaker , D. P. Shoemaker , Acta Crystallogr. 1965, 18, 900.

[22] G. A. Landrum , R. Hoffmann , J. Evers , H. Boysen , Inorg. Chem. 1998, 37, 5754.

[23] S. Cirafici , E. Franceschi , *J*. Less‐Common Met. 1979, 66, 137.

[24] G. O. Brunner , D. Schwarzenbach , Z. Kristallogr. 1971, 133, 127.

[25] G. H. Grosch , K. J. Range , J. Alloys Compd. 1996, 233, 30.

[26] R. Freccero , J. M. Hübner , Y.u. Prots , W. Schnelle , M. Schmidt , F. R. Wagner , U. Schwarz , Y.u. Grin , Angew. Chem., Int. Ed. 2021, 60, 6457. 10.1002/anie.202014284 PMC7986909 33236821

[27] MS1P e.V ., FHI‐aims, an all electron, numerically tabulated, atom‐centred‐orbital electronic structure code, 071914_7, Fritz‐Haber‐Institut, Berlin 2024.

[28] V. Blum , R. Gehrke , F. Hanke , P. Havu , V. Havu , X. G. Ren , K. Reuter , M. Scheffler , Comput. Phys. Commun. 2009, 180, 2175.

[29] R. F. W. Bader , Atoms in Molecules – A Quantum Theory, Vol. 22, Clarendon Press, Oxford 1994.

[30] F. R. Wagner , R. Freccero , Y.u. Grin , Acta Crystallogr. A 2025, in press. 10.1107/S2053273325002347 PMC12053493 40292699

[31] B. Silvi , C. Gatti , J. Phys. Chem. A 2000, 104, 947.

[32] A. Ormeci , H. Rosner , F. R. Wagner , M. Kohout , Y.u. Grin , J. Phys. Chem. A 2006, 110, 1100. 16420014 10.1021/jp054727r

[33] A. I. Baranov , M. Kohout , J. Comput. Chem. 2008, 29, 2161. 18432614 10.1002/jcc.20985

[34] Y. Grin , A. Savin , B. Silvi , in The Chemical Bond, Wiley, New York 2014, pp. 345–382.

[35] L. Agnarelli , Y. Prots , M. Schmidt , M. Krnel , E. Svanidze , U. Burkhardt , A. Leithe‐Jasper , Y. Grin , ChemistryOpen 2022, 11, e202200118. 35726898 10.1002/open.202200118 PMC9210927

[36] A. Savin , R. Nesper , S. Wengert , T. F. Fassler , Angew. Chem., Int. Ed. 1997, 36, 1809.

[37] D. A. Lang , J. V. Zaikina , D. D. Lovingood , T. E. Gedris , S. E. Latturner , J. Am. Chem. Soc. 2010, 132, 17523. 21090715 10.1021/ja107436n

[38] A. F. Al Alam , S. F. Matar , N. Ouaïni , M. Nakhl , Prog. Solid State Chem. 2008, 36, 192.

[39] S. F. Matar , Prog. Solid State Chem. 2010, 38, 1.

[40] X. J. Feng , Y.u. Prots , M. Bobnar , M. P. Schmidt , W. Schnelle , J. T. Zhao , Y.u. Grin , Chem.‐Eur. J. 2015, 21, 14471. 26291332 10.1002/chem.201501236

[41] H. Pauly , A. Weiss , H. Witte , Int. J. Mater. Res. 1968, 59, 414.

[42] G. Brauer , W. Haucke , Z. Phys. Chem. 1936, 33B, 304.

[43] STOE & C. GmbH , WinXPow, Darmstadt, Germany, 2003.

[44] G. M. Sheldrick , Acta Crystallogr. A. 2008, 64, 112. 18156677 10.1107/S0108767307043930

[45] G. M. Sheldrick , Acta Crystallogr. C. 2015, 71, 3.

[46] K. Honda , Ann. Phys. (Leipzig) 1910, 337, 1027.

[47] M. Owen , Ann. Phys. (Leipzig) 1912, 342, 657.

[48] F. R. Wagner , Y.u. Grin , in Comprehensive Inorganic Chemistry III (Third Edition), Elsevier, New York 2023, pp. 222–237.

[49] M. Kohout , DGRID 4.6‐5.0, Dresden, Germany, 2018–2021.

[50] F. R. Wagner , QTgeom, MPI‐CPfS, Dresden, Germany, 2021.

