# Supplementary material for: TiNiSi‐Type ALiAu (A = Ca, Sr, Ba, Eu, Yb) Compounds: Alternative Use of “Excess” Electrons
Source: Chemistry. 2025 Apr 7;31(25):e202500134. doi: 10.1002/chem.202500134 (PMC12057603; doi:10.1002/chem.202500134)
Supplement: Supplementary file 1 — Supporting Information [file CHEM-31-e202500134-s001.docx]

Supporting Information
©Wiley-VCH 2019
69451 Weinheim, Germany

TiNiSi-Type *A*LiAu (*A* = Ca, Sr, Ba, Eu, Yb) Compounds: Alternative Use of “Excess” Electrons

Peter Höhn,*^[a]^ Daniel Menéndez Crespo,^[a]^ Matej Bobnar,^[b]^ Primož Koželj,^[c]^ Mitja Krnel,^[a]^ Yurii Prots,^[a]^ Marcus Schmidt,^[a]^ Frank R. Wagner,^[a]^ and Yuri Grin^[a]^

[a] Drs. P. Höhn, D. Menéndez Crespo, M, Bobnar, P, Koželj, M. Krnel, Yu, Prots, M. Schmidt, F. R. Wagner, Prof. Yu. Grin

Max-Planck-Institut für Chemische Physik fester Stoffe

Chemische Metallkunde
 Nöthnitzer Str. 40 01187 Dresden Germany
 E-mail: peter.hoehn@cpfs.mpg.de

[b] On leave in
Jožef Stefan Institute
Jamova cesta 39
1000 Ljubljana, Slovenia

[c] On leave in
University of Ljubljana

Faculty of Mathematics and Physics

Department of Physics

Jadranska ulica 19

1000 Ljubljana, Slovenia

**Abstract:** New TiNiSi-type compounds *A*LiAu (*A =* Ca, Sr, Ba, Eu, Yb) were obtained in the form of mm-sized single crystals by high-temperature centrifugation-aided filtration (HTCAF) from lithium melt. They are the first examples of TiNiSi-type representatives containing Li and a transition metal. The metallic phases show paramagnetic (Ca) or diamagnetic (Sr, Ba, Yb) behavior or antiferromagnetic ordering below 19 K (Eu). A new structural description is based on a hexagonal close packing with *A* occupying all octahedral and Li occupying half of the tetrahedral voids in an ordered fashion. Chemical bonding analysis supports the structural description and reveals the formation of eight-atomic *stella-quadrangula* bonds pinned on the empty tetrahedral holes, a bonding picture known from elemental metals.

DOI: 10.1002/anie.2016XXXXX

.

Table of Contents

[Table of Contents 2](#_Toc193356109)

[Results and Discussion 4](#_Toc193356110)

[Octahedral and tetrahedral voids in *fcc*, *hcp* and TiNiSi type structures 4](#_Toc193356111)

[CaLiAu *vs.* CaLiSb: Brunner-Schwarzenbach plots of two TiNiSi type structures 4](#_Toc193356112)

[CaLiAu: is it possible to fill the void? 4](#_Toc193356113)

[**Figure S1.** a) Bärnighausen tree depicting all variations for ordered occupation in an undistorted *hcp* arrangement; b) – e) Arrangement of tetrahedral voids, tetrahedrally coordinated species and octahedrally coordinated species in a *hcp* arrangement; f) – i) Relation of the lattice parameters and atomic coordinates for TiNiSi type structures.. 5](#_Toc193356114)

[**Figure S2.** Relative changes of the lattice parameters, unit cell volume and distances *A–*Au of *A*LiAu vs ionic radius of *A* in *A*LiAu compounds. 6](#_Toc193356115)

[**Figure S3.** Distances *A–*Au and Li–Au as well as angles Li–Au–Li in the crystal structures of *A*LiAu. 6](#_Toc193356116)

[**Figure S4.** a) Coordination polyhedra indicating the first coordination sphere of Ca, Li, and Au in the crystal structure of CaLiAu; b) Coordination polyhedra from BS procedure as a function of interatomic distances for CaLiAu; c) Coordination polyhedra from BS procedure as a function of interatomic distances for CaLiSb. 7](#_Toc193356117)

[**Figure S5.** The *M*Li_4_Ca_6_ coordination polyhedra in the crystal structures of CaLiAu and CaLiSb. 8](#_Toc193356118)

[**Figure S6.** Band structure of CaLiAu. 8](#_Toc193356119)

[Magnetic susceptibility measurements 9](#_Toc193356120)

[Electrical resistivity measurements 9](#_Toc193356121)

[**Figure S7.** Electrical resistivity of *A*LiAu (*A* = Ca, Sr, Ba, Eu, Yb). 9](#_Toc193356122)

[Crystal Structures 10](#_Toc193356123)

[**Figure S8.** Low-angle parts of of measured and calculated X-ray powder patterns of YbLiAu, CaLiAu, EuLiAu, SrLiAu, and BaLiAu, indicating the absence of impurity phases. 10](#_Toc193356124)

[**Table S1.** Crystallographic data for *A*LiAu (*A* = Yb, Ca, Sr, Eu, Ba). 11](#_Toc193356125)

[**Table S2.1.** Atom sites and equivalent displacement factors for YbLiAu according to X-ray single crystal refinement 12](#_Toc193356126)

[**Table S3.1.** Anisotropic displacement factors of YbLiAu according to X-ray single crystal refinement. 12](#_Toc193356127)

[**Table S2.2.** Atom sites and equivalent displacement factors for CaLiAu according to X-ray single crystal refinement. 12](#_Toc193356128)

[**Table S3.2.** Anisotropic displacement factors of CaLiAu according to X-ray single crystal refinement. 12](#_Toc193356129)

[**Table S2.3.** Atom sites and equivalent displacement factors for SrLiAu according to X-ray single crystal refinement. 12](#_Toc193356130)

[**Table S3.3.** Anisotropic displacement factors of SrLiAu according to X-ray single crystal refinement. 12](#_Toc193356131)

[**Table S2.4.** Atom sites and equivalent displacement parameters for EuLiAu according to X-ray single crystal refinement. 13](#_Toc193356132)

[**Table S3.4.** Anisotropic displacement parameters of EuLiAu according to X-ray single crystal refinement. 13](#_Toc193356133)

[**Table S2.5.** Atom sites and equivalent displacement parameters for BaLiAu according to X-ray single crystal refinement. 13](#_Toc193356134)

[**Table S3.5.** Anisotropic displacement parameters of BaLiAu according to X-ray single crystal refinement. 13](#_Toc193356135)

[**Table S4.** Selected interatomic distances and angles in YbLiAu, CaLiAu, SrLiAu, EuLiAu, and BaLiAu. 13](#_Toc193356136)

[References 14](#_Toc193356137)

[Author Contributions 14](#_Toc193356138)

Results and Discussion

## Octahedral and tetrahedral voids in *fcc*, *hcp* and TiNiSi type structures

Both the cubic and hexagonal close-packed structures of *M* contain one octahedral *oct* and two tetrahedral *tet* voids per formula unit. While in the cubic case all regular variations of packing variants are known (e.g. NaCl (all octahedral voids occupied), CaF_2_ (all tetrahedral voids occupied), AlLiSi / Half Heusler (all octahedral and ordered half of the tetrahedral voids occupied) and Li_3_Bi / Heusler (all octahedral and all tetrahedral voids occupied), this is not the case for hexagonal close-packed structures.

Apart from NiAs and related phases (*hcp* with all octahedral voids (*oct*) occupied),^[1]^ the only other examples known are the ReB_2_ type structures,^[2]^ in which all tetrahedral voids (*tet*) are occupied. The complete occupation of all voids, both tetrahedral and octahedral, is not realized in any case in *hcp*; rather, in structures of Na_3_As type all tetrahedral voids as well as the common faces of adjacent octahedral voids are occupied.^[3]^

Total occupation of all octahedral voids and an ordered half occupation of the tetrahedral voids is possible in a *hcp* structure upon symmetry reduction. According to group-subgroup relations and as shown in the Bärnighausen tree (Figure S1a), four different order variants can be proposed for the ordered occupation of the tetrahedral voids by *tet* and □ in an idealized *hcp* arrangement (Figure S1b-S1e).

S1b) In subgroup *P*–3*m*1 (# 164), the empty tetrahedral voids are arranged in double layers in (001) with one half facing up and the other half facing down and both sharing edges. The *hcp* structure is built-up by polyhedra *Moct*_6_*tet*_4_, the empty tetrahedral voids are tetrahedrally surrounded by three *oct* and one *tet*. However, the centers of these tetrahedra □*M*_4_ (^2^/_3_, ^1^/_3_, ^1^/_8_) and □*oct*_3_*tet* (^2^/_3_, ^1^/_3_, ^3^/_32_) are not identical, but slightly apart.

S1c) In *P*6_3_*mc* (# 186), all tetrahedral voids face upwards and build a 3D network sharing common apices. Concerning the polyhedra *Moct*_6_*tet*_4_, the same applies as in S1b).

S1d) In *P*–6*m*2 (# 187), the tetrahedral voids form 1D strands via alternating common faces and corners which are connected by common apices to form a 3D network. The *hcp* structure is built-up by two different types of polyhedra, one polyhedron *Moct*_6_*tet*_6_ with *tet* located in (001), the other *Moct*_6_*tet*_2_ with *tet* located along [001]. In contrast to the other examples S1b, S1c, and S1e, the neighboring tetrahedral voids are located in a trigonal-prismatic cavity *Moct*_6_.

S1e) In *Pnma* (#62), strands of edge-sharing tetrahedral voids alternately facing up and down along [010] are observed, which are connected by common apices to form a 3D network. Concerning the polyhedra *Moct*_6_*tet*_4_, the same applies as in S1b).

To the knowledge of the authors, none of these ideal variants S1b-e) are realized in structural chemistry, however, as shown in Figures 3c,d) and S1f-i), the TiNiSi type ^[4]^ structure may be regarded as a highly distorted *hcp* arrangement of *M* (Si) with octahedral voids *oct* occupied by Ti and half the tetrahedral voids *tet* by Ni, the other half remaining empty □. The resulting coordination spheres around *M* are shown for the ideal case (Figure S1f), Ti in TiNiSi (Figure S1g), Sb in CaLiSb (Figure S1h), and Au in CaLiAu (Figure S1i).

In ZrBeSi,^[5]^ another 1:1:1 structure type derived from *hcp*, the octahedral voids and the common faces of neighboring tetrahedral voids are occupied, resulting in a trigonal-bipyramidal coordination for Be.

## CaLiAu *vs.* CaLiSb: Brunner-Schwarzenbach plots of two TiNiSi type structures

Characteristically for the TiNiSi family,^[4]^ the coordination polyhedra around Ca, Li and Au in CaLiAu are formed by 18 (6xCa, 6xLi, 6xAu), 12 (6xCa, 2xLi, 4xAu) and 9+1 (5+1xCa, 4xLi) atoms, respectively (Figure S4). Following the Brunner–Schwarzenbach formalism it is interesting to note, that the longest Ca–Au distance considered in the polyhedron around Ca (red circle in upper leftt graph in Figure S4a,b) does not belong to the first coordination sphere of Au, lying far *outside* the relevant range (red circle in upper right graph in Figure S4a,b). The same applies for the other compounds *A*LiAu (*A =* Yb, Eu, Sr, Ba).

Therefore, the coordination polyhedron around Au has to be regarded as a (distorted) capped square antiprism (Figure S5a). However, the resulting space in form of a tetragonal pyramid (semi-transparent area in Figure S5a) should not be considered as such a tetragonal pyramidal void, since any atom located in its center (~ 0.384, 1/4, 0.613) effectively sits in the center of an octahedron built-up by three Li, two Ca and one Au. Furthermore, it should be emphasized, that in isotypic semiconducting CaLiSb,^[6]^ as well as in all other *A*Li*T* (*A* = Ca, Sr, Eu, Yb; *T* = As, Sb, Bi), the coordination polyhedra and Brunner-Schwarzenbach plots show subtle, but significant differences compared to CaLiAu (Figure S4c,d, Figure S5b). In particular, in CaLiSb the Brunner-Schwarzenbach plots of Ca and Sb match, so the coordination spheres of antimony and calcium have six corresponding (Ca, Sb) neighbors each resulting in CN(Sb) = 10.

## CaLiAu: is it possible to fill the void?

Considering the crystal structure of CaLiAu as a packing of these AuLi_4_Ca_6_ bi-capped tetragonal antiprisms, the crystal structure contains one tetrahedral void per formula unit in 4*c* (~ 0.41, ¼, 0.065), with 1 Li and 3 Ca as nearest neighbors, as shown in Figure S5c. Via common edges, these Ca_3_Li tetrahedra form chains along [010]. Albeit small, we tried to introduce N (or H, for that matter) on this site in our quest for nitridometalates ^[7]^ and metalide nitrides,^[8]^ but did not succeed. In all experiments, phases *A*LiAu were received with neither any indication for a potential phase width nor (partial) occupation of this void.

In contrast, TbNiSnD ^[9]^ and CeNiSnH ^[10]^ (these phases should be rather labeled TbSnNiD and CeSnNiH, respectively, since Ni is located on the position of Si in TiNiSi) are the only examples for a filled TiNiSi type structure reported up to now. However, in these phases the site occupied by hydrogen and deuterium, respectively, is situated *within* the polyhedron NiSn_4_*RE*_6_ (gray tetrahedron in Figure S5d), whereas the tetrahedral site mentioned above remains empty.


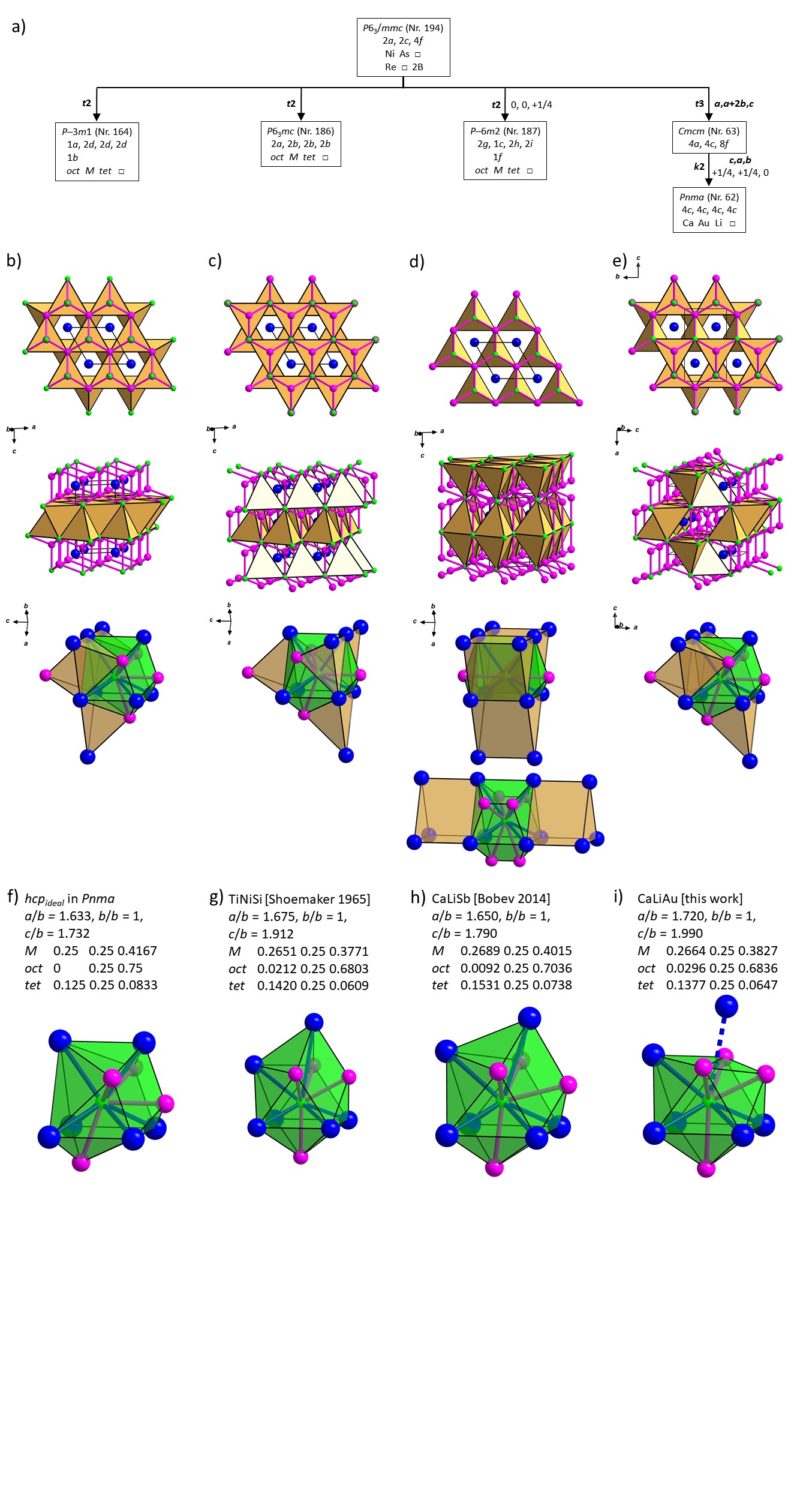


**Figure S1.** a) Bärnighausen tree depicting all variations for ordered occupation of the octahedral and half of the tetrahedral voids in an undistorted *hcp* arrangement; b) – e) Arrangement of tetrahedral voids (grey), tetrahedrally coordinated species (*tet,* blue) and octahedrally coordinated species (*oct*, yellow) in a *hcp* arrangement (*M*, red); top: view along [001], center: view along [010], bottom coordination polyhedron *Moct*_6_*tet*_4_□_4_; f) – i) top: Relation of the lattice parameters and atomic coordinates for TiNiSi type structures in f) an undistorted *hcp* arrangement, g) TiNiSi, h) CaLiSb, i) CaLiAu, bottom: the resulting coordination polyhedra in the same orientation and adjusted for scale.


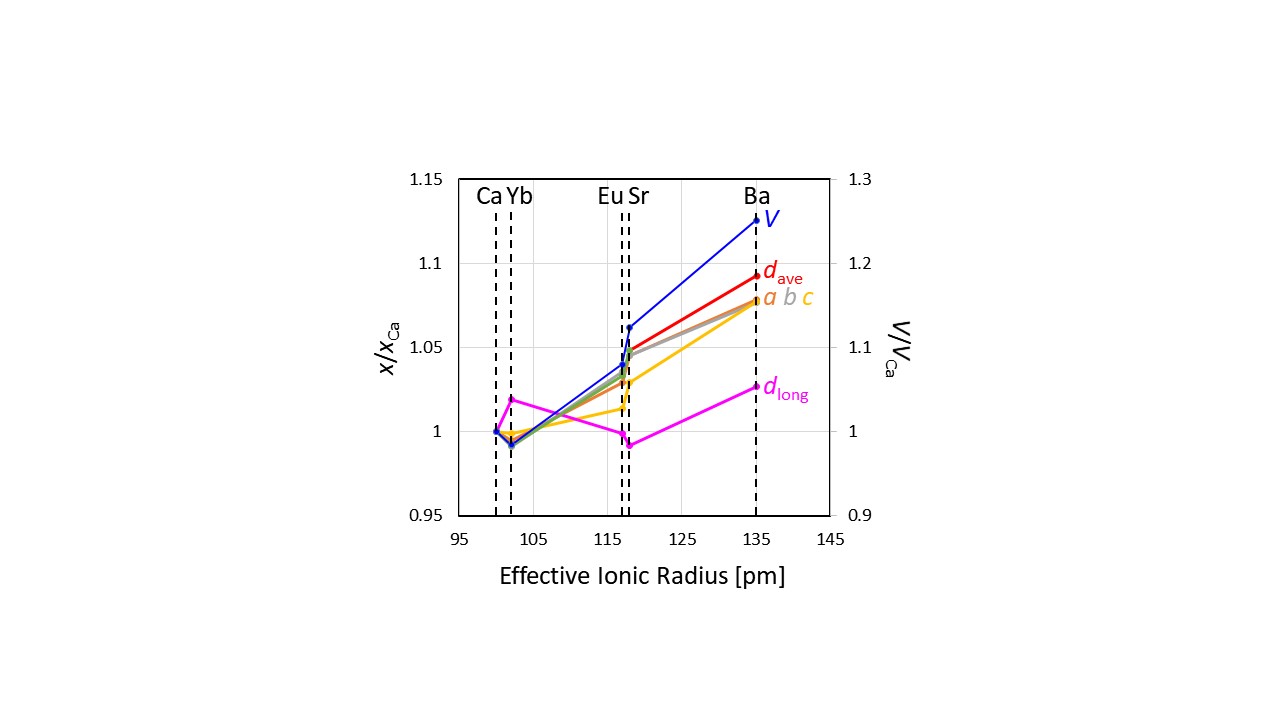


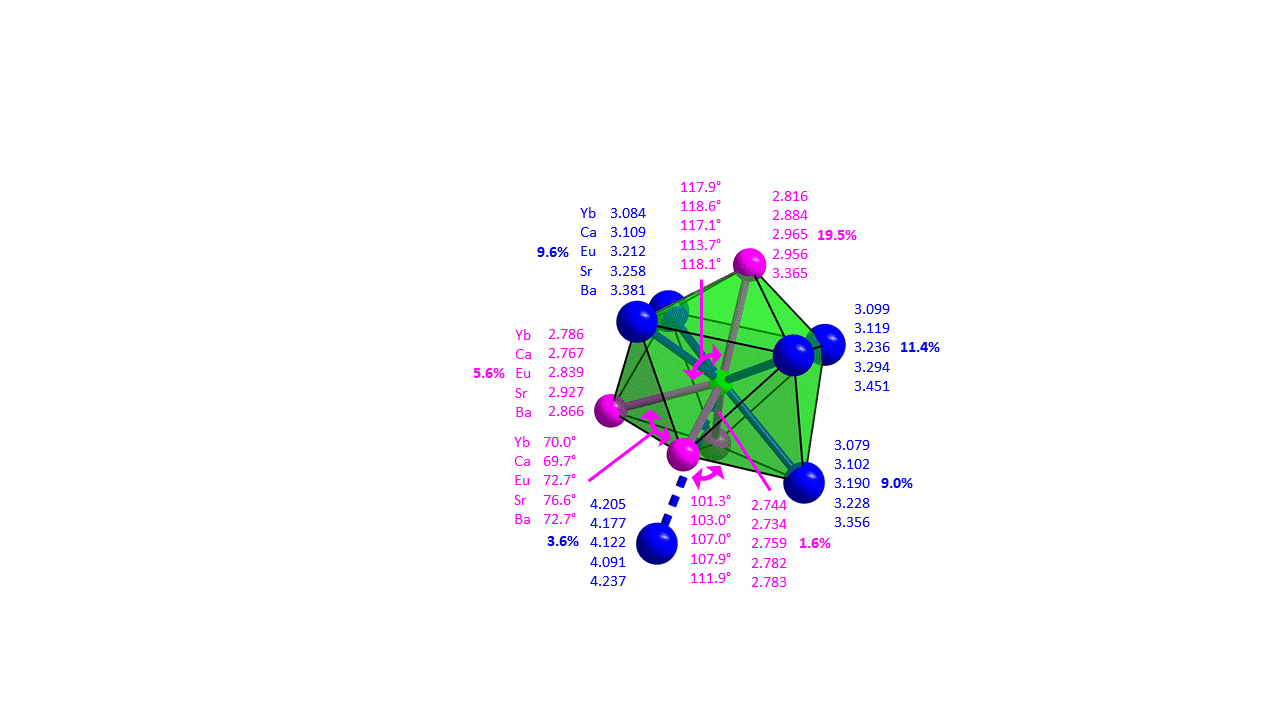
**Figure S2.** Relative changes of the lattice parameters, unit cell volume and distances *A–*Au of *A*LiAu vs ionic radius of *A* in *A*LiAu compounds.

**Figure S3.** Distances *A–*Au and Li–Au [Å] as well as angles Li–Au–Li [°] in the crystal structures of *A*LiAu.


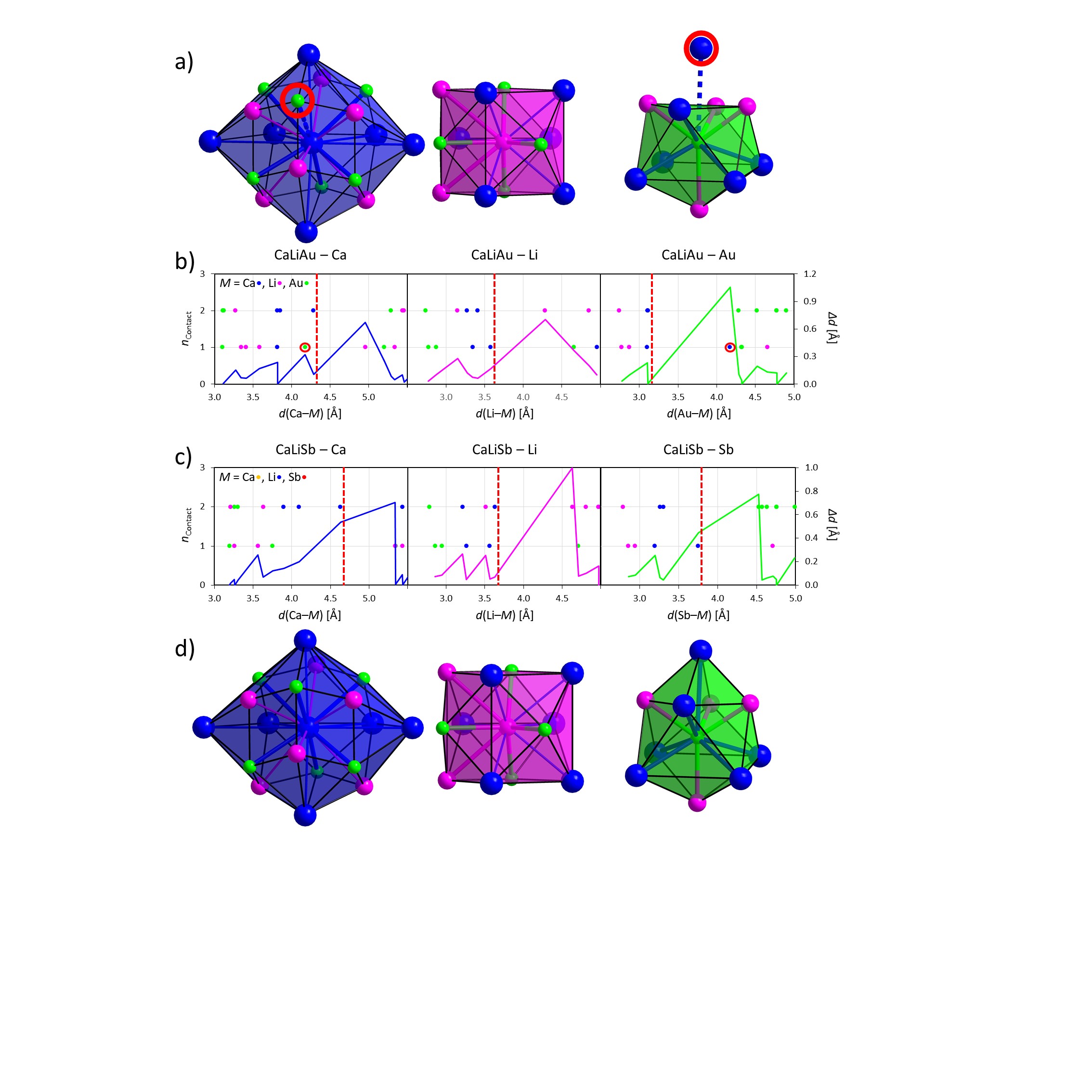


**Figure S4.** a) Coordination polyhedra indicating the first coordination sphere of Ca (yellow, left), Li (blue, center), and Au (red, right) in the crystal structure of CaLiAu; b) Coordination polyhedra from BS procedure as a function of interatomic distances for CaLiAu for Ca (left), Li (center), and Au (right); colored dots indicate number and type of contacts; the maximum of the distance-gap values (solid line) indicates the first neighbour outside the coordination polyhedron (dashed green line). It is evident, that some atoms (red circles) may belong to the coordination sphere of one atom, but not vice versa, resulting in the transparent-red tetragonal-pyramidal pseudo-void capping the coordination polyhedron around Au; c) Coordination polyhedra from BS procedure as a function of interatomic distances for CaLiSb for Ca (left), Li (center), and Sb (right); d) Coordination polyhedra indicating the first coordination sphere of Ca (yellow, left), Li (blue, center), and Sb (red, right) in the crystal structure of CaLiSb.


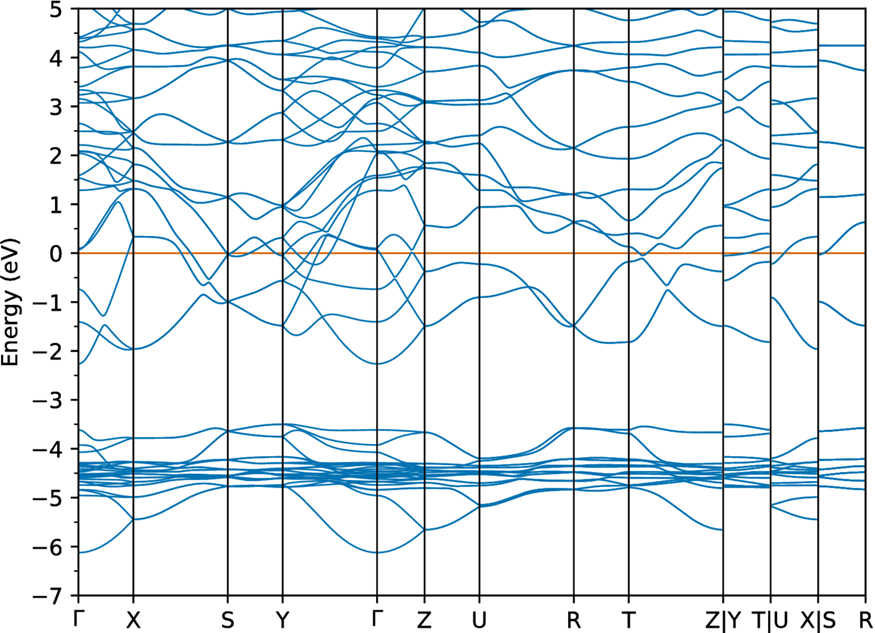

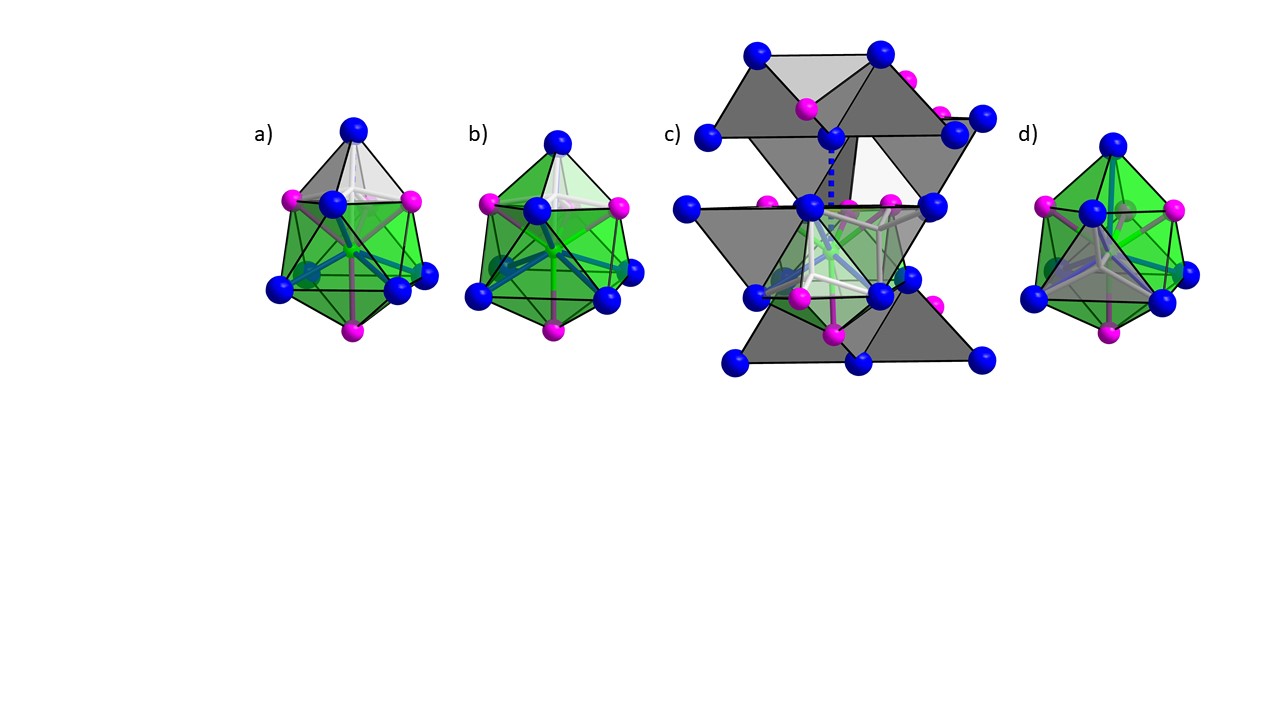
**Figure S5.** The *M*Li_4_Ca_6_ coordination polyhedra around a) *M =* Au (pseudo-void emphasized) and b) *M =* Sb in the crystal structures of CaLiAu and CaLiSb, respectively, c) Polyhedron *M*Li_4_Ca_6_ with surrounding tetrahedral voids Ca_3_Li emphasized in grey. These voids Ca_3_Li form chains via common edges along [010], and d) NiSn_4_Tb_6_ coordination polyhedron around Ni in the crystal structure of TbNiSnD (D in NiTb_3_ coordination emphasized, see text).

**Figure S6.** Band structure of CaLiAu.

**Magnetic susceptibility measurements**

The susceptibilities of *A*LiAu (*A =* Ca, Sr, Ba, Yb) are temperature independent above 60 K. The upturns at low temperatures are the result of small amounts of paramagnetic impurities or point defects. The equivalent concentrations of paramagnetic ions with spin *S* = 1/2 for CaLiAu, SrLiAu, BaLiAu and YbLiAu samples are 0.14, 0.14, 0.42 and 0.55 mol %, respectively.

The temperature independent magnetic susceptibilities *χ*_0_, are the sum of the core diamagnetism (*χ* _dia_) and of the Pauli paramagnetism (*χ* _Pauli_), as +65·10^–6^, –40·10^–6^, –55·10^–6^ and –57·10^–6^ emu/mol. The diamagnetic contributions of the cores for the ions Li^+^, Ca^2+^, Sr^2+^, Ba^2+^, Yb^2+^ and Au^3+^ taken from the literature to be –1·10^–6^ , –10.4·10^–6^, –19·10^–6^ , –26.5·10^–6^, –20·10^–6^ and –32·10^–6^ emu/mol,^[11]^ give a calculated *χ* _dia_ as –43.4·10^–6^, –52·10^–6^, –59.5·10^–6^ and –53·10^–6^ emu/mol. Therefore, the magnetic susceptibilities due to the conduction electrons (*χ* _Pauli_) are found to be 108.4·10^–6^, 12·10^–6^, 4.5·10^–6^ and 4·10^–6^ emu/mol. The uncertainty of *χ* _Pauli_ for CaLiAu is estimated to be about 20 %, while the uncertainties for SrLiAu, BaLiAu and YbLiAu are estimated to 100 % (or even more) due to the subtraction of two numbers of similar size. The resulting density of states at Fermi level are 3.3, 0.36, 0.14, and 0.12 states / (eV f.u.).

**Electrical resistivity measurements**

**Figure S7.** Electrical resistivity of *A*LiAu (*A* = Ca, Sr, Ba, Eu, Yb).

**Crystal Structures**

**
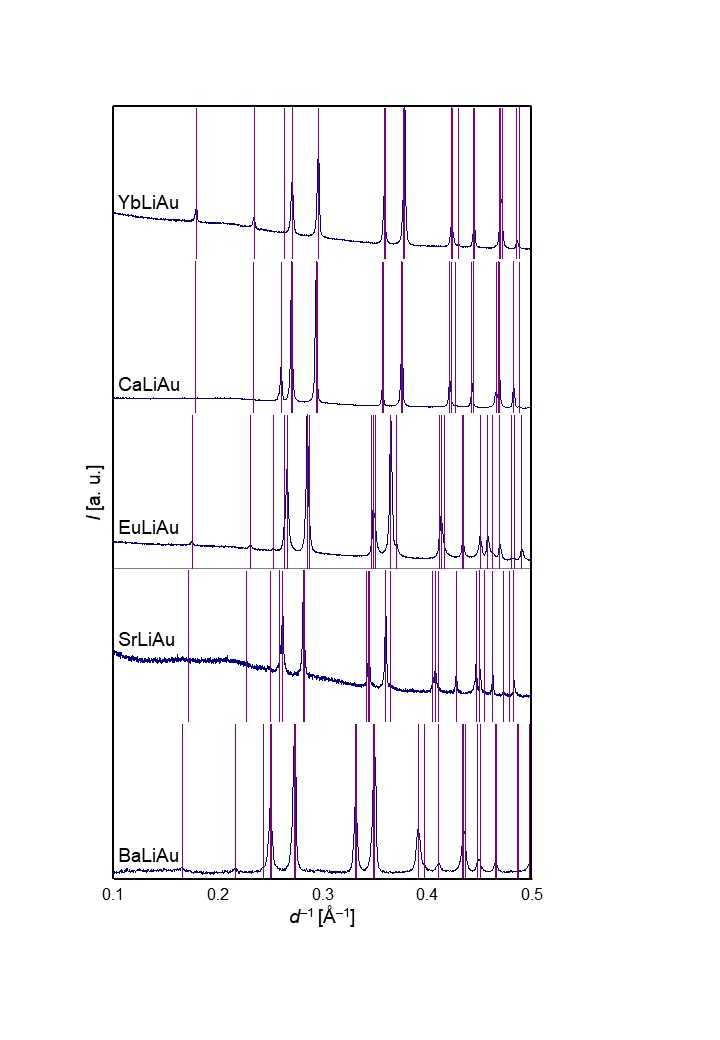
Figure S8.** Low-angle parts of measured (blue) and calculated (pink) X-ray powder patterns (Cu Kα) of YbLiAu (Cu Kα), CaLiAu (Cu Kα), EuLiAu (Co Kα), SrLiAu (Cu Kα), and BaLiAu (Cu Kα), indicating the absence of impurity phases.

**Table S1.** Crystallographic data for *A*LiAu (*A* = Yb, Ca, Sr, Eu, Ba).

| Composition | YbLiAu | CaLiAu | SrLiAu | EuLiAu | BaLiAu |
| --- | --- | --- | --- | --- | --- |
| Refinement method |  |  | Single crystal  SHELXL-2018^[12-13]^ |  |  |
| Crystal system |  |  | orthorhombic |  |  |
| Crystal color, shape |  |  | silver-metallic prism-like crystals |  |  |
| Space group |  |  | *Pnma* (No. 62) |  |  |
| *a* [Å]  *b* [Å]  *c* [Å] | 7.3283(12) 4.2451(7)  8.5129(13) | 7.3682(4)  4.2837(3)  8.5250(6) | 7.6923(10) 4.4982(5) 8.6873(11) | 7.5806(12) 4.4358(7) 8.6408(14) | 7.947(2)  4.6121(13)  9.184(3) |
| *V* [Å^3^] | 264.83(7) | 269.08(3) | 300.59(6) | 290.56(8) | 336.62(17) |
| *Z* |  |  | 4 |  |  |
| Molar mass [g⋅mol^–1^] | 376.95 | 243.99 | 291.53 | 355.87 | 341.25 |
| δ*_x_* [g⋅cm^–3^] | 9.454 | 6.023 | 6.442 | 8.135 | 6.734 |
| Temperature *T* [K] | 293 | 293 | 293 | 293 | 293 |
| Diffractometer |  |  | Rigaku AFC7 |  |  |
| Detector |  |  | CCD Saturn 724+ |  |  |
| Radiation, λ [Å] |  |  | Mo *K*α, 0.71073 |  |  |
| F(000) [*e*] | 608 | 408 | 480 | 580 | 552 |
| μ [mm^–1^] | 90.023 | 56.181 | 66.139 | 71.452 | 54.843 |
| Scan mode |  |  | ϕ |  |  |
| 2θ range [°] | 7.3–70.0 | 7.3–85.0 | 7.0–60.0 | 7.0–70.0 | 6.7–50.0 |
| *hkl* ranges | –10 ≤ h ≤ 11,  –2 ≤ k ≤ 6,  –13 ≤ l ≤ 13 | –13 ≤ h ≤ 13,  –3 ≤ k ≤ 8,  –12 ≤ l ≤ 16 | –10 ≤ h ≤ 10,  –6 ≤ k ≤ 2,  –12 ≤ l ≤ 10 | –12 ≤ h ≤ 11,  –7 ≤ k ≤ 3,  –12 ≤ l ≤ 13 | –9 ≤ h ≤ 8,  –3 ≤ k ≤ 5,  –10 ≤ l ≤ 7 |
| No. refl. | 3274 | 5423 | 2127 | 4935 | 905 |
| No. refl. unique | 619 | 998 | 487 | 705 | 330 |
| *R*_int_ | 0.0539 | 0.0399 | 0.0424 | 0.0433 | 0.0411 |
| Refined parameters | 17 | 20 | 20 | 19 | 16 |
| Extinction coeff.^*^ | 0.0037(9) | 0.0149(10) | 0.0035(9) | – | – |
| *R*1, *wR*2 (all data) | 0.052, 0.122 | 0.026, 0.0671 | 0.046, 0.103 | 0.026, 0.043 | 0.070, 0.161 |
| GOOF | 1.180 | 1.080 | 1.043 | 1.074 | 0.990 |
| Highest electron diff. peak [Å^–3^] | 4.32 | 4.47 | 2.86 | 1.37 | 5.37 |
| Deepest electron diff. hole [Å^–3^] | –9.52 | –4.09 | –7.21 | –1.86 | –3.49 |

^*^ 𝑘 [(1 + .001 𝑥 𝐹_𝑐_^2^ 𝜆^3^)/sin2𝜃]^–1/4^

**Table S2.1.** Atom sites and equivalent displacement factors for YbLiAu according to X-ray single crystal refinement

| Atom | Site | Occupancy | *x* / *a* | *y* / *b* | *z* / *c* | *U*_eq_ /Å^2^ |
| --- | --- | --- | --- | --- | --- | --- |
| Yb | 4*c* | 1 | 0.03180(8) | ¼ | 0.68192(7) | 0.0172(2) |
| Li | 4*c* | 1 | 0.140(3) | ¼ | 0.068(2) | 0.011(4) |
| Au | 4*c* | 1 | 0.26458(8) | ¼ | 0.38093(7) | 0.0186(2) |

**Table S3.1.** Anisotropic displacement factors *U*_ij_ /Å^2^ of YbLiAu according to X-ray single crystal refinement. Li was refined in isotropic approximation.

| Atom | *U*_11_ | *U*_22_ | *U*_33_ | *U*_23_ | *U*_13_ | *U*_12_ |
| --- | --- | --- | --- | --- | --- | --- |
| Yb | 0.0115(4) | 0.0178(3) | 0.0223(4) | 0 | -0.00146(19) | 0 |
| Au | 0.0134(3) | 0.0169(3) | 0.0254(3) | 0 | 0.00187(18) | 0 |

**Table S2.2.** Atom sites and equivalent displacement factors for CaLiAu according to X-ray single crystal refinement.

| Atom | Site | Occupancy | *x* / *a* | *y* / *b* | *z* / *c* | *U*_eq_ /Å^2^ |
| --- | --- | --- | --- | --- | --- | --- |
| Ca | 4*c* | 1 | 0.02969(11) | ¼ | 0.68351(9) | 0.01615(7) |
| Li | 4*c* | 1 | 0.1367(12) | ¼ | 0.0635(10) | 0.0188(14) |
| Au | 4*c* | 1 | 0.26640(2) | ¼ | 0.38262(2) | 0.01615(7) |

**Table S3.2.** Anisotropic displacement factors *U*_ij_ /Å^2^ of CaLiAu according to X-ray single crystal refinement.

| Atom | *U*_11_ | *U*_22_ | *U*_33_ | *U*_23_ | *U*_13_ | *U*_12_ |
| --- | --- | --- | --- | --- | --- | --- |
| Ca | 0.0143(3) | 0.0147(3) | 0.0157(3) | 0 | -0.0016(2) | 0 |
| Li | 0.020(4) | 0.018(3) | 0.019(3) | 0 | 0.003(3) | 0 |
| Au | 0.01624(10) | 0.01352(10) | 0.01869(10) | 0 | 0.00201(4) | 0 |

**Table S2.3.** Atom sites and equivalent displacement factors for SrLiAu according to X-ray single crystal refinement.

| Atom | Site | Occupancy | *x* / *a* | *y* / *b* | *z* / *c* | *U*_eq_ /Å^2^ |
| --- | --- | --- | --- | --- | --- | --- |
| Sr | 4*c* | 1 | 0.02214(17) | ¼ | 0.68882(16) | 0.0180(3) |
| Li | 4*c* | 1 | 0.159(4) | ¼ | 0.073(3) | 0.015(5) |
| Au | 4*c* | 1 | 0.27662(7) | ¼ | 0.39341(8) | 0.0205(3) |

**Table S3.3.** Anisotropic displacement factors *U*_ij_ /Å^2^ of SrLiAu according to X-ray single crystal refinement.

| Atom | *U*_11_ | *U*_22_ | *U*_33_ | *U*_23_ | *U*_13_ | *U*_12_ |
| --- | --- | --- | --- | --- | --- | --- |
| Sr | 0.0097(6) | 0.0228(6) | 0.0215(7) | 0 | -0.0022(5) | 0 |
| Li | 0.028(14) | 0.005(9) | 0.011(10) | 0 | 0.006(10) | 0 |
| Au | 0.0123(4) | 0.0214(4) | 0.0277(4) | 0 | 0.0044(2) | 0 |

**Table S2.4.** Atom sites and equivalent displacement parameters for EuLiAu according to X-ray single crystal refinement.

| Atom | Site | Occupancy | *x* / *a* | *y* / *b* | *z* / *c* | *U*_eq_ /Å^2^ |
| --- | --- | --- | --- | --- | --- | --- |
| Eu | 4*c* | 1 | 0.02452(4) | ¼ | 0.68709(4) | 0.01754(9) |
| Li | 4*c* | 1 | 0.1448(15) | ¼ | 0.0657(13) | 0.018(2) |
| Au | 4*c* | 1 | 0.27375(3) | ¼ | 0.38961(3) | 0.02005(8) |

**Table S3.4.** Anisotropic displacement parameters *U*_ij_ /Å^2^ of EuLiAu according to X-ray single crystal refinement.

| Atom | *U*_11_ | *U*_22_ | *U*_33_ | *U*_23_ | *U*_13_ | *U*_12_ |
| --- | --- | --- | --- | --- | --- | --- |
| Eu | 0.01548(14) | 0.01592(15) | 0.02122(17) | 0 | -0.00186(10) | 0 |
| Li | 0.030(6) | 0.010(4) | 0.013(5) | 0 | 0.009(4) | 0 |
| Au | 0.01768(12) | 0.01559(12) | 0.02689(15) | 0 | 0.00338(9) | 0 |

**Table S2.5.** Atom sites and equivalent displacement parameters for BaLiAu according to X-ray single crystal refinement.

| Atom | Site | Occupancy | *x* / *a* | *y* / *b* | *z* / *c* | *U*_eq_ /Å^2^ |
| --- | --- | --- | --- | --- | --- | --- |
| Ba | 4*c* | 1 | 0.02419(18) | ¼ | 0.68916(15) | 0.0293(5) |
| Li | 4*c* | 1 | 0.139(6) | ¼ | 0.055(5) | 0.042(10) |
| Au | 4*c* | 1 | 0.28215(12) | ¼ | 0.39976(12) | 0.0351(5) |

**Table S3.5.** Anisotropic displacement parameters *U*_ij_ /Å^2^ of BaLiAu according to X-ray single crystal refinement. Li was refined in isotropic approximation due to the limited data available (cf. Table S1).

| Atom | *U*_11_ | *U*_22_ | *U*_33_ | *U*_23_ | *U*_13_ | *U*_12_ |
| --- | --- | --- | --- | --- | --- | --- |
| Ba | 0.0222(8) | 0.0273(8) | 0.0385(9) | 0 | -0.0029(5) | 0 |
| Au | 0.0254(7) | 0.0257(6) | 0.0543(8) | 0 | 0.0073(4) | 0 |

**Table S4.** Selected interatomic distances (Å) and angles (°) in YbLiAu, CaLiAu, SrLiAu, EuLiAu, and BaLiAu.

| Distance | *n* | YbLiAu | CaLiAu | SrLiAu | EuLiAu | BaLiAu |
| --- | --- | --- | --- | --- | --- | --- |
| Au–Li | 2 | 2.7442(114) | 2.7339(53) | 2.7816(156) | 2.7594(67) | 2.7827(259) |
| Au–Li | 1 | 2.7856(219) | 2.7674(88) | 2.9269(266) | 2.8388(114) | 2.8662(477) |
| Au–Li | 1 | 2.8159(176) | 2.8835(86) | 2.9556(307) | 2.9651(113) | 3.3646(461) |
| Au–*A* | 1 | 3.0785(9) | 3.1019(8) | 3.2280(15) | 3.1899(6) | 3.3564(19) |
| Au–*A* | 2 | 3.0836(7) | 3.1089(6) | 3.2575(11) | 3.2124(4) | 3.3807(14) |
| Au–*A* | 2 | 3.0986(7) | 3.1186(6) | 3.2942(11) | 3.2360(4) | 3.4510(14) |
| Au–*A* | 1 | 4.2052(10) | 4.1767(8) | 4.0911(16) | 4.1221(7) | 4.2371(21) |
| Angles | *n* (cf. Figure 4) | YbLiAu | CaLiAu | SrLiAu | EuLiAu | BaLiAu |
| Li–Au–Li | 2 | 69.984(471) | 69.728(256) | 76.554(659) | 72.743(267) | 72.718(1097) |
| Li–Au–Li | 1 [010] | 101.336(474) | 102.950(257) | 107.907(678) | 106.970(271) | 111.935(1113) |
| Li–Au–Li | 1 (101) | 117.897(465) | 118.596(250) | 113.672(642) | 117.069(258) | 118.098(1004) |
| Li–Au–Li | 2 | 129.168(468) | 128.337(252) | 126.046(662) | 126.463(262) | 123.940(1022) |

# References

[1] G. Aminoff, *Z. Kristallogr.* **1923**, *58*, 203 - 219.

[2] S. J. La Placa, B. Post, *Acta Crystallogr.* **1962**, *15*, 97-99.

[3] G. Brauer, E. Zintl, *Z. Phys. Chem.* **1937**, *37B*, 323-352.

[4] R. Freccero, Yu. Grin, F. R. Wagner, *Dalton Trans.* **2023**, *52*, 8222-8236.

[5] J. W. Nielsen, N. C. Baenziger, *Acta Crystallogr.* **1954**, *7*, 132-133.

[6] M. C. Schäfer, N.-T. Suen, S. Bobev, *Dalton Trans.* **2014**, *43*, 16889-16901.

[7] P. Höhn, R. Niewa, in *Handbook of Solid State Chemistry, Part 1. Materials and Structure of Solids*, Wiley-VCH, **2017**, pp. 251-359.

[8] M. Pathak, D. Stoiber, M. Bobnar, A. Ovchinnikov, A. Ormeci, R. Niewa, P. Höhn, *Z. Anorg. Allg. Chem.* **2017**, *643*, 1557-1563.

[9] V. A. Yartys, R. V. Denys, O. Isnard, R. G. Delaplane, P. Svedlindh, K. H. J. Buschow, *J. Magn. Magn. Mater.* **2007**, *311*, 639-643.

[10] R. A. Klein, R. Balderas-Xicohténcatl, J. P. Maehlen, T. J. Udovic, C. M. Brown, R. G. Delaplane, Y. B. Cheng, R. V. Denys, A. J. Ramirez-Cuesta, V. A. Yartys, *J. Alloys Compd.* **2022**, *894*, 162381.

[11] G. A. Bain, J. F. Berry, *J. Chem. Educ.* **2008**, *85*, 532-536.

[12] G. M. Sheldrick, *Acta Crystallogr. A* **2008**, *64*, 112-122.

[13] G. M. Sheldrick, *Acta Crystallogr. C* **2015**, *71*, 3-8.

# Author Contributions

P. H. planned and carried out the material synthesis. Yu. P. and P. H. planned and carried out the single crystal X-ray diffraction measurements and analyzed the data. M. B., P. K., and M. K. performed physical property measurements on single crystals and microcrystalline powders. M. S. performed DTA/TG investigations. F.R.W., D. M. C., and Yu. G. performed quantum chemical calculations and bonding analysis. All authors contributed in writing the manuscript. P. H. and Yu. G. supervised the project.
